# Genetic Rescue of X-Linked Retinoschisis Mouse (*Rs1*^−/y^) Retina Induces Quiescence of the Retinal Microglial Inflammatory State Following AAV8-*RS1* Gene Transfer and Identifies Gene Networks Underlying Retinal Recovery

**DOI:** 10.1089/hum.2020.213

**Published:** 2021-07-16

**Authors:** Camasamudram Vijayasarathy, Yong Zeng, Matthew J. Brooks, Robert N. Fariss, Paul A. Sieving

**Affiliations:** ^1^ Section for Translational Research in Retinal and Macular Degeneration,; ^2^ Neurobiology-Neurodegeneration and Repair Laboratory, and; ^3^ Biological Imaging Core, National Eye Institute, National Institutes of Health, Bethesda, Maryland, USA; ^4^ Department of Ophthalmology, Center for Ocular Regenerative Therapy, School of Medicine, University of California at Davis, Sacramento, CA, USA.

**Keywords:** X-linked retinoschisis, RNA-seq, AAV8-retinoschisin, microglia activation, immune quiescence, gene therapy

## Abstract

To understand *RS1* gene interaction networks in the X-linked retinoschisis (XLRS) mouse retina (*Rs1*^−/y^), we analyzed the transcriptome by RNA sequencing before and after *in vivo* expression of exogenous retinoschisin (*RS1*) gene delivered by AAV8. RS1 is a secreted cell adhesion protein that is critical for maintaining structural lamination and synaptic integrity of the neural retina. *RS1* loss-of-function mutations cause XLRS disease in young boys and men, with splitting (“schisis”) of retinal layers and synaptic dysfunction that cause progressive vision loss with age. Analysis of differential gene expression profiles and pathway enrichment analysis of *Rs1*-KO (*Rs1*^−/y^) retina identified cell surface receptor signaling and positive regulation of cell adhesion as potential *RS1* gene interaction networks. Most importantly, it also showed massive dysregulation of immune response genes at early age, with characteristics of a microglia-driven proinflammatory state. Delivery of AAV8-*RS1* primed the *Rs1*-KO retina toward structural and functional recovery. The disease transcriptome transitioned toward a recovery phase with upregulation of genes implicated in wound healing, anatomical structure (camera type eye) development, metabolic pathways, and collagen IV networks that provide mechanical stability to basement membrane. AAV8-*RS1* expression also attenuated the microglia gene signatures to low levels toward immune quiescence. This study is among the first to identify *RS1* gene interaction networks that underlie retinal structural and functional recovery after *RS1* gene therapy. Significantly, it also shows that providing wild-type *RS1* gene function caused the retina immune status to transition from a degenerative inflammatory phenotype toward immune quiescence, even though the transgene is not directly linked to microglia function. This study indicates that inhibition of microglial proinflammatory responses is an integral part of therapeutic rescue in XLRS gene therapy, and gene therapy might realize its full potential if delivered before microglia activation and photoreceptor cell death. Clinical Trials. gov Identifier NTC 02317887.

## Introduction

X-linked retinoschisis (XLRS) is an inherited form of macular degeneration seen in young boys and men and caused by loss-of-function mutations in the retinoschisin (*RS1*) gene.^1,2^ XLRS patients suffer extensive structural schisis (splitting) of the outer plexiform and inner nuclear layers (INLs) of the neurosensory retina.^3–5^ Disorganization of the inner retinal layers disrupts the synaptic signal transmission from photoreceptors to ON-bipolar cells and leads to slowly progressive loss in vision.^6,7^ The electroretinogram (the retinal electrical response to light stimulation) recorded in XLRS shows the bipolar b-wave is reduced relative to the photoreceptor a-wave and is widely used for clinical diagnosis of XLRS.^8,9^ Mouse models that recapitulate human XLRS clinical symptoms (*Rs1*^−/y^ and *Rs1*-KO) helped delineate the disease pathophysiology and allowed assessing *in vivo* therapeutic benefits of AAV-mediated *RS1* gene therapy for XLRS.^10–13^ AAV8-mediated *RS1* gene expression in mouse model of XLRS lead to the closure of the schisis cavity, restoration of the retinal architecture, and functional improvement to synaptic visual signaling. Clinical trials are underway to evaluate the safety, tolerability, and efficacy of intravitreal application of an AAV vector expressing human *RS1* gene in XLRS patients.^14^

The *RS1* gene encodes retinoschisin (RS1), a secreted protein that has a crucial role in maintaining cellular organization of the retina.^15^ Both retinal photoreceptors and bipolar cells express and secrete RS1 into the extracellular space.^16^ RS1 protein encodes a conserved discoidin domain sequence motif (DS), also known as F5_8-type C domain sequence. DS domain-containing proteins are involved in cellular adhesion, migration, cell proliferation, and blood coagulation (Coagulation Factors V and VIII).^2^ Based on this structural involvement, the schisis pathology found in XLRS patient retinas has been attributed to RS1 loss of function as a cell adhesion protein.^2^ Cryo-transmission electron microscopy revealed a double octamer of assembled mature RS1 in solution, with 16 RS1 monomers arranged in two back-to-back octameric rings and into extensive branched networks.^17–19^ Although the paired octamer rings and branched networks of RS1 suggest a cell–cell adhesion structure that links adjacent cells, no *in vivo* evidence exists that RS1 can link two neighboring cells. Studies indicated that retinal Na^+^/K^+^-ATPase, consisting of subunits Atp1a3 and Atp1b2, is essential for anchoring RS1 to plasma membranes and identified the glycosylated Atp1b2 subunit as the direct interaction partner for RS1.^20,21^ Recent studies showed that RS1 regulates Erk signaling and apoptosis in retinal cells, while also exerting regulatory effects on Na^+^/K^+^-ATPase signaling and localization.^22,23^

Despite advances in understanding XLRS pathophysiology,^6,15,24,25^ the factors underlying the heterogeneity of clinical phenotypes in terms of disease onset, symptoms, severity, and progression, as seen even between family members, have not been entirely clear. Although nearly 200 pathogenic *RS1* mutations have been reported, only a rough genotype–phenotype correlation has been established.^8,26–29^ A microarray-based genome-wide expression profiling of the *Rs1*-KO retina had identified adhesion, cytoskeleton, vesicular trafficking, and immune response as key pathways affected in early postnatal *Rs1*-KO mice.^30^ Microarray technology has several limitations for dynamic range, specificity, and sensitivity, and we chose to utilize RNA sequencing (RNA-seq) for a comparative transcriptome analyses between wild-type (WT) mouse and the *Rs1*-KO retinas before and after treatment. We sought to identify gene networks that mediate disease biology (loss of function) and define gene regulatory networks underlying structural and functional recovery following AAV8-mediated *RS1* gene expression (gain of function). We provide here the first in-depth characterization of *RS1* gene interaction networks that underlie retinal structural and functional recovery after *RS1* gene therapy. Significantly, it also shows that providing wild-type *RS1* gene function caused the retina immune status to transition from a degenerative inflammatory phenotype toward immune quiescence, even though the transgene is not directly linked to microglia function.

## Materials and Methods

### Experimental mice

Since the *Rs1* gene is on the X chromosome and expresses as a classical X-linked condition, we used only male mice in this study. Retinoschisin knockout (*Rs1*^−/y^; Rs1-KO; XLRS) male mice and age-matched littermate C57BL/6 WT male mice were generated in our laboratory,^11^ and were studied at 14–60 days in postnatal age (P). The *Rs1*-KO mice have been maintained at National Institutes of Health (NIH, Bethesda, MD) since 2004 and backcrossed onto the WT C57BL/6J line (Jackson Laboratory, Bar-Harbor, ME) for >25 generations. We described the phenotype and natural history previously.^31^ This research was conducted in accordance with the Association for Research in Vision and Ophthalmology Statement on the Use of Animals in Ophthalmic and Vision Research and was overseen by the Animal Care and Use Committee of the National Eye Institute (Protocol Number NEI-617) under NIH Institutional Biosafety Committee-approved Human Pathogen Registration Document #4766 and Recombinant DNA Registration Document RD-09-II-03.

### Experimental design

Twenty-two *Rs1*-KO (*Rs1*^−/y^) mice were divided into eight groups based on their genotype and treatment regimen (*n* = 3–4 mice per group), with six WT male mice as needed. They represented four transcriptome pairs. The experimental design is shown in Table 1.

**Table 1.** Experimental design

Experiment	Transcriptome Pair	Postnatal Day Age	Number of Mice	Genotype	Treatment SRI*^a^*on P14	Retina Collection
1	1	P12	3	WT	**—**	P12
1	1	P12	3	*Rs1*-KO	**—**	P12
1	2	P21	3	WT	**—**	P21
1	2	P21	3	*Rs1*-KO	**—**	P21
2	3 (C7)	P14	4	*Rs1*-KO	AAV8-Null	P21
2	3 (G7)	P14	4	*Rs1*-KO	AAV8-*RS1*	P21
2	4 (C35)	P14	4	*Rs1*-KO	AAV8-Null	P49
2	4 (G35)	P14	4	*Rs1*-KO	AAV8-*RS1*	P49

^a^ SRI—bilateral subretinal injection on postnatal day 14 (P14). Two retinas from the same mouse were pooled for RNA isolation. Retinas from three or four mice per group were used as biological replicates.

WT, wild type.

### AAV8 vectors

Self-complementary (sc) AAV8-scRS/IRBPhRS (AAV8-*RS1*) delivered human retinoschisin (*RS1*) cDNA under control of an *RS1* promoter and interphotoreceptor binding protein (IRBP) enhancer, was produced at the Children's Hospital of Philadelphia, (Philadelphia, PA; GMP Lot # A8FP1 A1407C).^14^ Vector was diluted to 2–12 viral genomes (vg)/mL with excipient, and 1 μL of vector (2–9 vg/eye) was injected subretinally in anesthetized *Rs1*-KO male mice at P14. As negative controls, other *Rs1*-KO mice received 1 μL of AA8-Null vector (2–9 vg/eye), which has the AAV8 capsid, ITR from AAV2, CMV promoter, but no transgene (produced by Vector Biolabs, Malvern, PA).

### Gene delivery by subretinal Injection

Mouse was anesthetized with ketamine, 80 mg/kg, and xylazine, 10 mg/kg given intraperitoneal, and one drop of 0.5% tetracaine was applied topically to the cornea. Under a dissecting microscope, the treated eye was positioned cornea upward, and the conjunctiva membrane was removed with scissors and forceps. A 33-gauge sharp needle was used to make a small hole 1 mm from the temporal limbus down to the subretinal space, and a blunt 35-gauge needle was advanced through this hole to inject 1 μL of vector. Triple antibiotic ophthalmic ointment was applied to the injection site.

### RNA extraction, mRNA-seq library preparation, and sequencing

Eyes were enucleated at prescribed time points and dissected to isolate the retina. Total RNA was extracted according to QIAzol lysis-based reagent miRNA Mini Kit (Qiagen, Inc., Germantown, MD). Qualitative and quantitative analysis of extracted RNA samples were carried out with a 2100 Bioanalyzer Nano Chip (Agilent Technologies Genomics, Santa Clara, CA). RNA integrity number of the RNA samples used for RNA Seq ranged between 8.4 and 9.4, and RNA samples were stored at −80°C. Strand-specific mRNA-sequencing libraries were constructed from 100 ng of total RNA with the TruSeq Stranded mRNA Library Prep Kit (Illumina, San Diego, CA). RNA libraries were sequenced on the Illumina HiSeq2500 platform to produce over 60 million 76 nucleotide paired-end reads for Experiment-1 and 30 million 150 nucleotide paired-end reads for postinjection day 7 and 35 samples in Experiment 2. The number of sequencing reads generated for each sample and their alignment statistics are shown in Supplementary Table S1. RNA-seq analysis pipeline is depicted in Supplementary Fig. S1A. Sequencing reads passing Illumina's chastity filter were used for further analysis.^32^ Illumina adapter, poly-T and poly-A read trimming was performed with Trimmomatic v0.36. Quality control of trimmed Fastq files was assessed using FastQC v0.11.4 (https://bioinformatics.babraham.ac.uk/projects/fastqc/) software. Reads were aligned to the genome for quality control evaluation using STAR v2.5.1b (PMID: 23104886) employing the two-pass transcriptome-guided protocol with the ENCODE options, with GRCm38.p4 and Ensembl v84 annotation (*Mus musculus*/house mouse genome assembly) genome reference files downloaded from Ensembl FTP. Transcript-level quantitation was performed using Kallisto v0.43.0 (PMID: 27043002) employing a merged transcript cDNA and ncRNA FASTA file downloaded from the Ensembl ftp site. Gene-level quantitation was obtained using Tximport v1.2.0 (PMID: 26925227) with the “lengthScaledTPM” option in the R programming environment v3.3.0 (https://r-project.org/). Gene-level results were used for the remaining analysis. Gene-level counts were trimmed mean of M values normalized in edgeR v3.16.5 (PMID: 20196867).

### Differential expression analysis

Differential expression (DE) analysis for experiment 1 (*Rs1*-KO vs. WT at P12 and P21) was performed in pairwise comparisons separately using Limma v3.30.12. Only genes having >1 counts-per-million (CPM) in all replicates of each comparison group were kept for DE analysis. Dispersion estimation was executed with the voom function and contrast statistics with the eBayes function by specifying all coefficients for analysis. Genes having >2-fold change and false discovery rate (FDR) of 1% were kept for further analysis. Hierarchical clustering performed on DE genes employed Ward's method using Euclidean distance on *Z*-scores from the averaged log2 CPM values. Functional gene enrichment was performed using topGO v2.24.0 (PMID: 16606683). Reduced redundancy of gene ontology (GO) results was performed using the runTest function with Fisher's exact test. DE analysis for experiment 2 (*Rs1*-KO-AAV8-RS1 (G) vs. *Rs1*-KO-AAV8-null (C) on day 7 and 35 post-injection) was performed in pairwise comparisons separately. Only genes having >1 CPM value in all replicates of any comparison group were kept for DE analysis. DE analysis between experimental samples and controls used the exact test in edgeR v3.20.9 (PMID: 17728317). Genes having >1.5-fold change and FDR of 5% were kept for further analysis. Functional gene analysis was performed using the gProfiler v0.6.6 (PMID: 27098042) package in R with gene sets from GO biological process (BP) and Reactome databases. All raw and processed data are available through National Center for Biotechnology Information Gene Expression Omnibus (https://ncbi.nlm.nih.gov/geo/): GSE153874.

### Quantitative real-time polymerase chain reaction analysis for the validation of RNA-seq data

Total RNA was extracted using TRIzol reagent-based miRNeasy Mini Kit (Qiagen, Inc.) using manufacturer's instructions. The RT^2^ Easy First Strand Kit (Qiagen, Inc.) was used for first-strand cDNA synthesis and genomic DNA elimination in RNA samples. About 2 μg of total RNA was used for cDNA synthesis with random hexamers according to the manufacturer's protocol. Three microliters of cDNA was used as a template, and each PCR reaction (20 μL) was performed on the QuantStudio™ 6 and 7 Flex Real-Time PCR System (Thermo Fisher Scientific, Waltham, MA) with TaqMan^®^ probes (Supplementary Table S2). The manufacturer's default thermal cycling conditions were followed (40 cycles of 1 s at 95°C and 20 s at 60°C). Reactions were run in triplicate in three independent experiments. 18s rRNA was amplified as endogenous control (for the internal reference). The results were expressed as *n*-fold induction or inhibition in gene expression relative to endogenous control calculated using the ΔΔCT method. Data were processed using QuantStudio 6 and 7 Flex Real-Time PCR System Software.

### Optical coherence tomography image collection and analysis

Optical coherence tomography (OCT) images were obtained with the Envisu R2200 SD-OCT Ophthalmic Imaging System (Bioptigen, Inc., Durham, NC) as described previously.^33^ Animals were anesthetized and placed in a custom holder after dilating the pupils. Radial volume scans consisting of four B-scans (1,000 A-scans per B-scan) were collected at 45° angular intervals with an average of five frames each. Rectangular volume scans with 100 B-scans (1,000 A-scans per B-scan) were collected from the retinal pigment epithelium (RPE) to the posterior lens. Cavity size, outer nuclear layer (ONL) thickness, outer retina reflective band number, and changes in retinal structure were evaluated.

### Immunohistochemistry

Retinas were processed for immunohistochemistry (IHC) as previously described.^33^ Whole eyes were enucleated from WT and from treated and untreated *Rs1*-KO mice after euthanasia. Eye globes were fixed for half hour in 4% paraformaldehyde in sodium phosphate buffer on ice, and eyecup was made by removal of anterior parts of eye and then fixed in same fixation buffer on ice for another 90 min. The eyecups were then cryoprotected in 30% sucrose overnight at 4°C before being embedded in optimal cutting temperature compound (Sakura Finetek USA, Inc., Torrance, CA). Sagittal retinal cryosections (10 μm) were cut using research cryostat (Leica Biosystems, Buffalo Grove, IL). Retinal sections were permeabilized in a low Triton X-100 (0.1%) containing phosphate-buffered saline (PBS, PBST). Supplementary Table S2 lists antibodies and other reagents used in this study. Nonspecific binding sites in the tissue were blocked with 20% normal serum in PBS from the same host species as the labeled secondary antibody. Then primary antibodies with appropriate dilution in the serum containing PBST buffer were added: Rs1 (1:1,000); Iba1 (1:500); and Cd68 (1:400). Subsequently, the retinal tissues or sections were washed three times with PBST for 10 min each time. Fluorescent secondary antibodies were diluted 1:1,000 in PBST and added to retinal sections for 1 h. The nuclei were stained with 4′,6′-diamidino-2-phenylindole in washing buffer and sections were mounted with Fluorogel (Electron Microscopy Sciences, Hatfield, PA). Retinal images were captured and processed using a Nikon C2 confocal microscope with Advance Element software (Nikon, Tokyo, Japan). Image analysis was performed by using image-editing software (Photoshop CS6; Adobe System, Inc.).

### Statistics

Prism GraphPad software (8.4.3. for Windows; GraphPad Software, San Diego, CA) was used for statistical analysis. Changes in expression levels were evaluated between treatment groups by one-way analysis of variance and confirmed by unpaired *t*-test and two-stage step-up method of Benjamini, Krieger, and Yekutieli. We considered *p* < 0.05 as threshold for significance. The data shown are representative of two or three independent experiments performed in triplicate.

## Results

### Evaluation of *Rs1*-KO (*Rs1*^−/y^) retina

This study used RNA-seq to identify RS1-dependent molecular changes within the retina that precede and coincide with photoreceptor/synaptic functional loss. In the *Rs1*-KO mouse retina, retinal layer formation and synaptic protein expression in the outer plexiform layer (OPL) are normal up to postnatal day 14 (P14), and both structural schisis and synaptic dysfunction become apparent only after synapses have formed, P17–P21.^34,35^ In accordance with this, the schisis led to the upregulation of astrocyte marker glial fibrillary acidic protein (Gfap). Gfap is constitutively expressed in astrocytes in nerve fiber layer (NFL), but GFAP upregulation response to retinal injury is most pronounced in Muller cells.^36,37^ We analyzed retinas of *RS1*-KO mice at P12 (apparently normal) and P21 (coinciding with synaptic and structural changes) relative to age-matched WT controls.

### The sequencing and transcriptome data

RNA-seq analysis pipeline is depicted in Supplementary Fig. S1A. Supplementary Table S1 shows the number of sequencing reads generated for each sample and their alignment statistics. Gross gene expression differences between samples were visualized by principal component analysis using normalized, log2 CPM (gene-level CPM mapped reads) values of protein-coding genes. In Experiment 1 (Supplementary Fig. S1B), samples were segregated by age P12 versus P21 (PC1, 52.1% variance) and genotype *Rs1*-KO versus WT (PC2, 19.1% variance). In Experiment 2 (Supplementary Fig. S1C), principal components segregated the samples by age (PC1, 59.5% variance), treatment type (PC2, 17.4% variance), and treatment duration (PC3, 6.3% variance), respectively.

### Differential gene expression in *Rs1* KO (*Rs1*^−/y^) mice retina at P12 and P21

**Figure 1.**
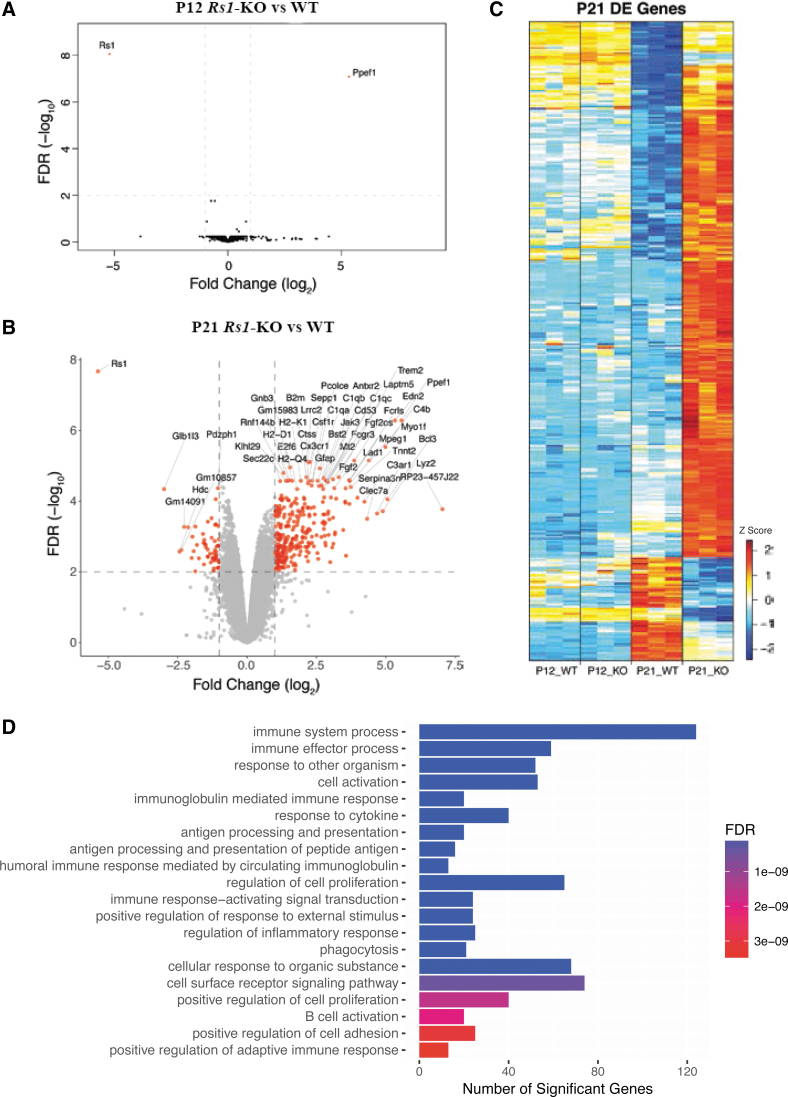
Transcription profiles of *Rs1*-KO (*Rs1*^−/y^) retina. **(A)** Volcano plot of the significantly differentially expressed genes (DEGs) (*red dots*) in the analysis of *Rs1*-KO (*Rs1*^-/y^) mouse retina relative to WT males at P12. **(B)** Volcano plot of the significantly *DEGs* (*red dots*) in the analysis of *Rs1*-KO mouse retina relative to WT retina samples at P21. Significance thresholds being fold change ≥2 and FDR <1% (dashed lines). Gene labels indicate those having fold change ≥2. **(C)** Hierarchical cluster analysis of gene expression patterns in WT and *Rs1*-KO mice retinas at ages P12 and P21 respectively. Hierarchical clustering of genes and samples was performed. A total of 12 retina RNA samples (3 samples each P12 WT, P12 *Rs1*-KO; p21 WT & P21 *Rs1*-KO) were sorted by hierarchical clustering and the data are presented in a matrix format; each row represents a particular cDNA and each column is an individual RNA sample. Number of expressed genes and their corresponding transcripts at each in vivo retina age using different low-level expression limit cutoffs: fold change ≥2 for any comparison and with a FDR <1%. Genes were clustered based on their Z-score value. **(D)** Most significantly enriched pathways of differentially expressed genes in *Rs1*-KO mice retina as analyzed by Gene Ontology Biological Processes. Number of differentially expressed genes in each pathway: P21-*Rs1*-KO vs. P21-WT mouse retinas showing 2-fold changes in gene expression levels. *p* = 0.01. FDR, false discovery rate.

DE analysis was performed using the Bioconductor packages, Limma, as detailed in “Materials and Methods.”^32^ Analysis of *Rs1*-KO versus WT retina identified two genes differentially expressed at P12, while 358 genes were differentially expressed at P21. When ordered by statistical significance (*p* 0.01- and two-fold change), the data showed 301 genes upregulated and 57 genes downregulated in P21 KO retina. The 57 downregulated genes showed no pathway enrichment. Supplementary Tables S3 and S4 show the complete list of differentially expressed genes (DEGs) at P21 and the significant enrichment of their GO terms. Except for two genes that were differentially expressed (*Rs1* and *Ppef1*), no other significant difference in gene expression profiles was found between *Rs1*-KO versus WT at P12. Volcano plots show the global transcriptional changes in *Rs1*-KO versus WT retinas at P12 and P21 (Fig. 1A, B). The heat map of DEGs (with ≥2-fold change for any comparison, and an FDR <1%) in four sample groups with three biological replicates each is shown in Fig. 1C and Supplementary Fig. S2. Genes were clustered based on their *Z*-score. The heat map also reveals age-dependent gene expression changes in WT retinas (P12WT vs. P21WT). The significant differences noted in P21 *Rs1*-KO retina indicated that they occurred early in the disease process and were relatively stable. To validate RNA-seq results, we analyzed nine DEGs by quantitative real-time polymerase chain reaction (qRT-PCR) to confirm genes for expression changes in the same set of mouse retinas as used in the RNA-seq analysis and in a second independent set of mice retinas. We observed statistically significant increases in *Edn2*, *B2m*, *Icam-1*, *Ppef1*, *Lad1*, *Stat3*, and *C4b* and a statistically significant decrease in *Cldn7* and *Zan*, concordant with the RNA-Seq analysis (Supplementary Fig. S3).

#### Immune system

P14–P16 is the time when *Rs1*-KO retina first shows histopathological features of structural schisis (splitting) in the inner retinal layers and disruption of OPL. The substantial transcriptome profile changes observed in *Rs1*-KO retina at P21 reflect the cumulative effects of a gliotic state and morphological changes that are underway at P14–P16. Functional enrichment analysis of upregulated genes in *Rs1*-KO retina revealed that the enriched module genes were mostly involved in immune system processes (Supplementary Table S4). The top 20 GO terms are shown in bar chart based on BP and ranked by fold enrichment (Fig. 1D). Supplementary Table S5 shows selected list of upregulated genes in *Rs1*-KO retina transcriptome at P21 with statistical significance (*p*-value) and magnitude of change (log twofold change) values. The transcriptome profile of *Rs1*-KO retina at P21 revealed upregulation of several microglia specific genes *Cx3cr1*, *Cd33*, *Gpr34*, *Fcrls*, *P2ry12*, *P2ry13*, *ApoE*, and *Trem2* (Supplementary Table S5). Early microglia activation is a hallmark of early photoreceptor degeneration. Other transcripts included adhesion molecules, extracellular matrix (ECM) proteins, and transcription factors, regulating microglia phenotype and immune responses. The strongest upregulated genes were *Bcl3*, *Ppef1*, *Edn2*, *Clec7a*, *Tlr7*, *Fgf2*, *C4b*, and *C3ar1* (8 to 37-fold increase). Most of the transcripts partially overlapped with sets of transcripts identified in genetically based photoreceptor diseases, including XLRS and acute photoreceptor injuries.^30,38^

### Consequences of AAV8-*RS1* expression

**Figure 2.**
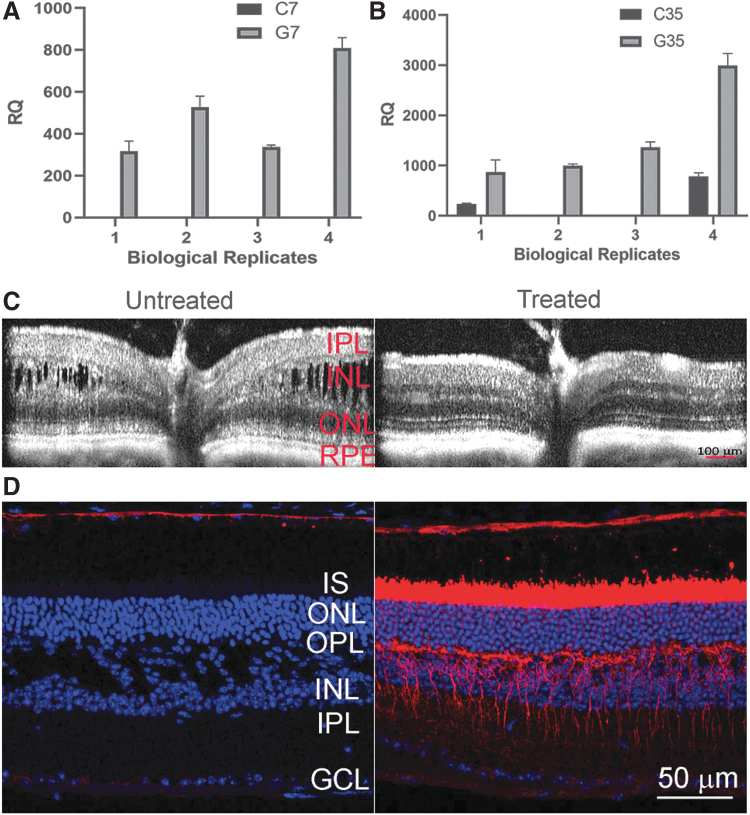
AAV8-delivered *transgene RS1* expression restores neural cell adhesion and retina lamination in *Rs1*-KO (*Rs1*^−/y^) retina. **(A-B)** qRT-PCR reaction confirmation of *RS1* gene expression in *Rs1*-KO (*Rs1*^-/y^ ) mice retina receiving subretinal injections of AAV8-*RS1* or AAV8-Null vector. 18s rRNA was amplified as endogenous control. The assay conditions are described in detail in Materials and Methods. The data are represented as a fold change in relative levels of indicated gene expression compared to those in WT control retina. **(A)** Exogenous *RS1* gene expression levels at post injection day 7, G7 and at **(B)** post injection day 35, G35. The relative quantity of RS1 mRNA was calculated using the delta-delta Ct method, considering the PCR signal of the target gene transcript of each sample, normalized to 18S rRNA relative to that of the control sample. The data was expressed as Mean ± SEM. **(C)** SD-OCT scans collected from *Rs1*-KO mice before (left panel) and 35 days after (right panel) *RS1* gene transfer. The scan of untreated eye showed large cavities splitting the OPL and INL with blurring of the margin between these two layers. In contrast, treated eyes showed much more organized retinal laminar structure: the size of cavities was reduced, the margin of OPL and INL became more distinct, and ONL thickness was partially preserved. **(D)** RS1 expression in uninjected *Rs1*-KO mice retina (left panel) and AAV8-*RS1*–injected retina (right panel) at 5 weeks after treatment (P49). Retinal sections were immunolabeled with anti-RS1 antibody (dilution 1:1000) and nuclei were counterstained with DAPI (*blue*). Gene transfer into *Rs1*-KO mice retina lead to strong expression of RS1 protein (*red*) in inner segments (IS) of photoreceptors and OPL and along bipolar cell processes, similar to that seen in WT mouse retina. The untreated retinas from *Rs1*-KO mice showed no RS1 labeling and displayed schisis cavities and bipolar cell layer disorganization. Scale bars: 50 μm. *n* = 3.

We performed transcriptome profiling of XLRS mouse retinas after application of AAV8-*RS1* vector (2–9 vg/eye) by subretinal injection on P14, to identify *RS1*-responsive genes. The control group received AAV8-null vector (2–9 vg/eye) at P14, and we evaluated the effects 7 and 35 days after vector application. Gene expression changes directly related to transgene *RS1* expression in *Rs1*-KO retina imply gain-of-function responses. We use the terminology G7 for Gain-of-Function at postinjection day 7 versus control (C7), and G35 versus C35 when studied at postinjection day 35. We first assessed the outcome of AAV8-*RS1* gene transfer using qRT-PCR to measure relative RNA abundance after AAV8-*RS1* gene transfer. *RS1* transgene expression was observed by 7 days postinjection (Fig. 2A), and a further twofold to fourfold increase in transgene expression levels occurred by 35 days postinjection (Fig. 2B). Figure 2C exemplifies SD-OCT scans collected from *Rs1*-KO mice before (left panel) and 35 days after (right panel) *RS1* gene transfer. An SD-OCT line scan of untreated eyes showed large cavities splitting the OPL and INL with blurring of the margin between these two layers. ONL thickness was markedly reduced. In contrast, treated eyes showed much more organized retinal laminar structure: the size of cavities was reduced, the margin of OPL and INL became more distinct, and ONL thickness was partially preserved. Protein expression assessed by IHC showed robust RS1 expression in photoreceptor and bipolar cell layers with a distribution similar to WT retina (Fig. 2D).

**Figure 3.**
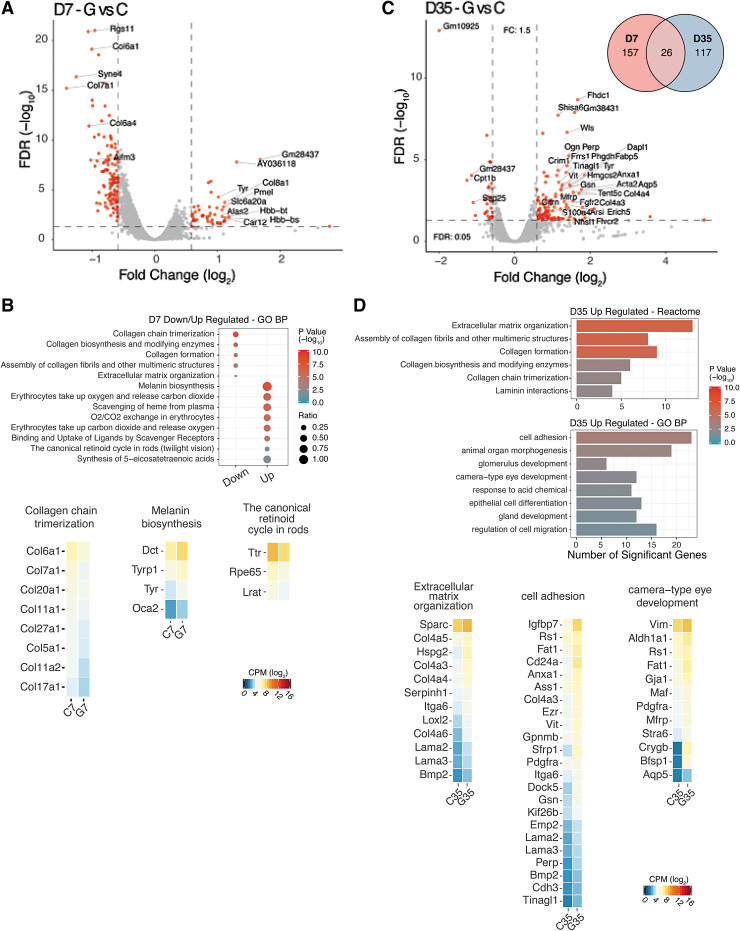
AAV8-*RS1* induced gene expression changes in *Rs1*-KO (*Rs1*^−/y^) retina. **(A)** Volcano plot of the significantly DEGs (*red dots*) in the analysis of *Rs1*-KO AAV8-RS1(G7) mouse retina relative to *Rs1*-KO AA8-Null retina (C7) samples at 7 days post-injection. Significance thresholds being fold change ≥1.5 and FDR <1% (*dashed lines*). Gene labels indicate those having fold change ≥1.5. **(B)** GO functional enrichment analysis for the down-regulated and up-regulated DEGs. Ratio is the proportion of each pathway's genes obtained in a particular group of the DEG list. *p*-value shows the significance in -log10 values. Expression heatmaps of selected marker genes for collagen chain trimerization, melanin biosynthesis and canonical retinoid cycle in rods. Expression values are represented as log2 CPM values. **(C)** Volcano plot of the significantly DEGs (*red dots*) in the analysis of *Rs1*-KO AAV8-RS1 (G35) mouse retina relative to *Rs1*-KO AA8-Null (C35) retina samples at 35 days post-injection. Significance thresholds being fold change ≥1.5 and FDR <1% (*dashed lines*). Gene labels indicate those having fold change ≥1.5. Inset shows Venn diagram of DEGs in different comparisons G vs. C. The overlapping portions of the different circles represent the number of DEGs common to these comparison groups. **(D)** Pathway enrichment analysis: Gene ontology biological processes (BP) and reactome pathways, the number of upregulated and significant genes. Expression heatmaps of selected marker genes for ECM organization, cell adhesion, camera type eye development in the retina. Expression values are represented as log2 CPM values.

DE analysis of *Rs1*-KO retinas expressing RS1 transgene 7 days postinjection (G7 vs. C7; AAV8-*RS1* vs. AAV8 Null) indicated that 183 genes were differentially expressed out of 12,686 annotated genes. Volcano plot shows the magnitude and the degree of differential gene expression resulting from exogenous *RS1* gene expression as early as 7 days after AAV8-*RS1* vector application (Fig. 3A). Fifty-seven genes were upregulated and 127 genes downregulated. At postinjection day 35 (G35 vs. C35), 143 of 12,673 annotated genes were differentially expressed (Fig. 3C), with 114 upregulated and 30 downregulated. For selected genes, DE fold change was confirmed by qRT-PCR (Supplementary Fig. S4). The complete lists of DEGs are shown in Supplementary Tables S6 and S7, respectively. Supplementary Table S8 shows a selected list of DEGs. At 7 days postinjection, transcriptome analysis showed downregulation of apoptosis-inducing factor mitochondria associated 3 (*Aifm3*, Supplementary Table S8), a proapoptotic protein that acts through mitochondrial depolarization, cytochrome *c* release, and caspase-3 activation.^39^ A few minor fibril-forming collagens (*Col5a1*, *Col11a1*, and *Col27a1*), fibril anchoring collagens (*Col7a1*), and fibril-associated collagens (*Col 6a1*, *Col6a4*, and *Col 20a1*) were downregulated (Fig. 3B and Supplementary Table S8). Mutations in these collagen genes lead to severe eye problems, including Stickler syndrome, Marshall syndrome (*Col 11*), and Sjogren's syndrome (*Col 5a1*), characterized by nearsightedness, retinal tearing, retinal detachment, keratoconus, blue sclera, and dry eye. However, none of these overlap the XLRS phenotype. *Col17a1* encodes a transmembrane protein, which plays a critical role in maintaining the linkage between intracellular and extracellular structural elements and is involved in epidermal adhesion and interacting components of the network-forming collagens, laminins, and some integrin types.

Of particular interest is the upregulation of genes involved in cycling retinoids between the photoreceptors and RPE: the 11-cis retinal-regenerating enzymes and retinol-transporting enzymes Rdh5, Rbp1, Rpe65, Stra6, and Lrat.^40,41^ The *Ttr* gene encodes for transthyretin that transports vitamin A (retinol) and thyroxine hormone throughout the body (Fig. 3B and Supplementary Table S8). Rbp1 is the carrier protein involved in retinol (vitamin A) transport from the liver storage site to peripheral tissue. Interestingly, melanogenic gene expression was upregulated. Melanin production is controlled by the tyrosinase (*Tyr*) gene, which regulates melanin expression in both the eye and the skin. Other *Tyr*-related genes included *Oca2* (P protein, also known as melanocyte-specific transporter protein), *Dct* (Dopachrome Delta-Isomerase), and *Tyrp1* (tyrosinase-related protein1). Other genes that were significantly upregulated included oxygen transport genes (*Hba*-alpha globin and *Hbb*-beta globin) and antioxidant paraoxonases (*Pon*1 and *Pon*3), which degrade lipid peroxides.

Comparison analysis revealed that the ECM and basement membrane genes dominated the list of altered genes in XLRS-*RS1* retina at 35 days postinjection (Fig. 3D). Basement membrane-associated genes *Col4a1*, *Col4a2*, *Col4a3*, *Col4a4*, *Col4a5 and Col4a6*, and *Col7a1* were upregulated (Fig. 3D and Supplementary Table S8), as also were laminins (*Lama2* and *Lama3*). Gene pathways inferred among upregulated genes included the following: focal adhesion, anatomical structure formation, wound healing, tissue development, canonical retinoid cycle, and camera-type eye development genes (Fig. 3D).

**Figure 4.**
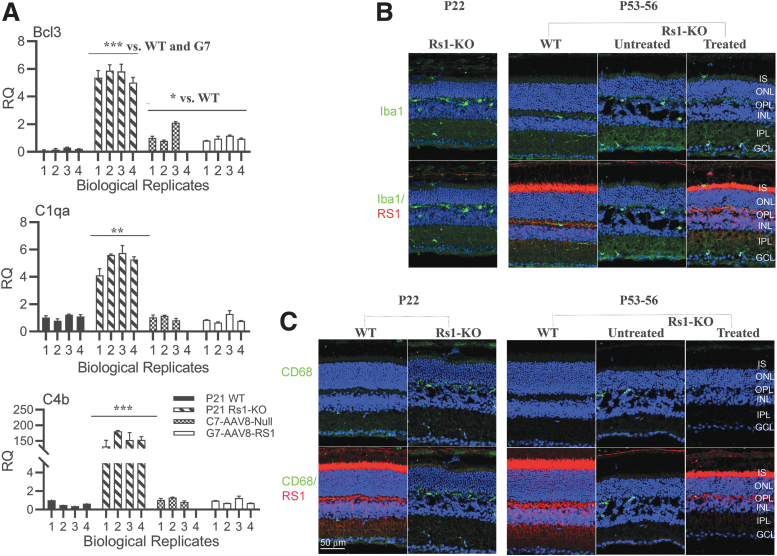
AAV8-*RS1* attenuates inflammatory responses and restores immune quiescence state in *Rs1*-KO (*Rs1*^−/y^) retina. **(A)** Exogenous *RS1* gene expression in *Rs1*-KO (*Rs1*^-/y^) retina suppresses pro- inflammatory C1qa, C4b, andBcl3 gene expression. Bar graphs of RNA expression for indicated genes in WT, *Rs1*-KO, C7 and G7 retinas. Experiment is described in Fig 2A. Data are expressed as Mean ± SEM. Statistical significance determined using the Holm-Sidak method, with alpha = 0.05. Each row was analyzed individually, without assuming a consistent SD. *p ≤ 0.05, **p ≤ 0.01, ***p ≤ 0.001. **(B)** Exogenous *RS1* gene expression inhibits microglial activation in *Rs1*-KO retina: Retinal sections double stained Iba (1:500, green) and Rs1 (1:1000, red). Microglia in age matched WT retinas showed a tiny cell soma, little perinuclear cytoplasm, and a small number of fine, branched processes covered in numerous projections. In the untreated *Rs1*-KO retinas at P21 and P49 an increase in microglial cell number was found compared with WT control retinas (Figure 1B). Moreover, numerous amoeboid Iba1 positive cells were observed in all retinal layers, with greater presence in IPL and the OPL. In retinas expressing exogenous *RS1* gene amoeboid Iba1 -positive cells were less abundant than in untreated in all layers of the retina and were mainly distributed in more internal GC layer. **(C)** Retinal sections double stained for RS1(1:1000, red) and CD68 (1:400, green). CD68 is a phagocytic marker associated with the involvement of monocytes/macrophages. CD68 staining is undetectable in age matched WT retinas and CD68 staining is intense in OPL in untreated *RS1*-KO retina. CD68 staining barely detectable in treated retina. Data from one representative experiment of two or three independent experiments. Scale bars: 50 μm. *n* = 3.

Of importance, exogenous AAV8-*RS1* expression in XLRS mouse retina efficiently attenuated the expression of proinflammatory genes. Immune response-specific signatures that dominated the untreated *Rs1*-KO retina transcriptome (Supplementary Table S5) were absent after AAV8-*RS1* expression. Most of the components in complement signaling *C4b*, *C1qa*, and proinflammatory/apoptosis markers (*Bcl3*) that were highly upregulated in the *Rs1*-KO retina were downregulated following exogenous *RS1* gene expression (Fig. 4A). AAV vectors (AAV capsid, CpG-containing AAV genome) were shown to trigger immune responses in mice,^42,43^ and it is rather surprising that the immune response transcripts are dampened by the administration of AAV8-null control vector (Fig. 4A). Following receptor-mediated endocytosis of virus, the endosomal pattern-recognizing toll-like receptors (Tlrs 2, 3, 7, 8, and 9) recognize viral nucleic acids and activate antiviral immune response mediated by the release of type I interferons (IFNs) followed by induction of a T helper 1-mediated adaptive immune response. Myd88 plays an essential role in inducing B cells capable of differentiating into antibody-secreting cells. Tlr9 was found to be critical in the activation of proinflammatory genes and forming adaptive response following AAV vector administration. AAV vectors also trigger nuclear factor-kappa B-dependent production and release of cytokine and chemokines. The immune responses were also found to be specific to strandedness (single strand, double strand, and sc). Most often, the proinflammatory cytokine responses to AAV vectors were transient and returned to baseline soon. These responses were mostly studied in liver and muscle gene transfer studies. In our previous study, intravitreal injection of AAV8-*RS1* in the mouse did not cause any significant inflammatory response at either 2e9 or 2e10 vg/eye and we did not detect antibodies to viral capsid or transgene.^44^ In AAV8-*RS1* gene therapy clinical trial, ocular inflammation was observed in patients receiving high vector doses (1e10 and 1e11 vg). Systemic antibodies against AAV8 capsid increased in a dose-related manner, but no antibodies against retinoschisin protein were observed.^14^ Only one patient showed IFNγ producing T cells against AAV8 capsid coinciding with ocular inflammation, but his T cell response tapered off by day 60.

We checked immunoreactivity for Iba1 in cryosections of P53-56 mouse retina. Figure 4B shows confocal micrographs of WT and of treated and untreated *Rs1*-KO mice retinal sections double labeled for RS1 (red) and Iba1 (green). Iba1is a microglia/macrophage marker. In normal WT mouse retina, microglia were limited to the inner and outer plexiform and ganglion cell layers (GCLs) and possessed small cell soma with numerous ramified projections (Fig. 4B). Since upon activation, microglia become less ramified and increase their soma size, these cell morphology changes are indicative of a change in microglia activation state. In response to neuronal degeneration and gliosis, microglia assume an activated phenotype characterized by an ameboid morphology and by migratory and phagocytic behavior.^45^ Iba1-positive cells with amoeboid morphology were scarce in WT retinas. Iba1 (green) labeling was increased in *Rs1*-KO retina when compared to WT control, implicating microglial activation. Many amoeboid microglial cells, with short and thick primary and terminal processes and enlarged soma, were observed in all retinal layers of *Rs1*-KO retinas at P22 and P53–P56. The density of microglial cells in each region of the AAV8-*RS1* injected retina was lower than in untreated *Rs1*-KO retinas, and the microglia mainly distributed in the GCL. Figure 4C shows the confocal microscopy images of mouse retina sections double stained for Rs1 and CD68, an inflammation marker associated with monocytes/macrophages. Relative to age-matched controls, many infiltrating CD68+ cells were present in the OPL region of *Rs1*-KO retina, both at P22 and P53–56. CD68 staining was absent in treated retina expressing exogenous *RS1*. These results imply that provision of exogenous *RS1* by AAV8-*RS1* caused the inflammatory phenotype of the *Rs1*-KO retina to transition toward a state of immune quiescence.

## Discussion

We used RNA-seq to explore changes in the gene expression profiles in the XLRS mouse (*Rs1*-KO) retina caused by “loss-of-*Rs1* gene function” and then looked at the “gain-of-function” transcriptome response following the expression of exogenous *RS1* gene by delivery of AAV8-RS1 vector. The *Rs1*-KO retina showed dysregulation of both innate and adaptive immune response genes coinciding with development of retinal splitting. Furthermore, AAV8-*RS1* delivery restored neural cell adhesion and retina lamination in *Rs1*-KO retina and was accompanied with attenuated inflammatory response gene expression and upregulation of photoreceptor, RPE, and ECM genes linked to RS1 protein localization and retinal function.

### Transcriptome signatures reveal rapid induction of microglia-specific genes

Transcriptome analysis of *Rs1*-KO versus WT retinas exhibited few differences in gene expression at P12 when the inner retina has just formed and when photoreceptors are beginning to elaborate inner and outer segments. By P21, all retinal cells layers are present and functioning, and retinal development is essentially complete soon after. The large-scale changes in microglia and inflammatory response gene profiles in *Rs1*-KO at P21 reflect cellular responses to retinal injury associated with splitting of retinal layers, the formation of intraretinal cavities, gliotic changes, microglia activation, and photoreceptor cell death unfolding between P12 and 21.^30,34,35^ Although inflammatory responses during retinal pathophysiology triggered by microglia are known to be conserved,^38^ a recent single-cell RNA sequencing study in *Arr*(−/−) mouse model revealed that specific molecular phenotypes of immune cells and subpopulations likely depend on the tissue, type, and extent of neuronal degeneration.^46^ Studies also showed that alterations of ECM physical properties have dynamic effect on immune cell behavior and inflammation. It is likely that inflammatory responses and ECM alterations resulting from loss-of-RS1 function become intricately intertwined and trigger a cascade of signaling events that may impact retina structure and function and accelerate retina degeneration.^47–49^ There is growing evidence that retinal degenerative diseases activate microglia, which then play a pivotal role in propagating retinal degenerative processes.^45,50,51^ Upon activation, the microglia transition to an ameboid phenotype, which allows them to migrate from the inner retina to the outer plexiform and nuclear layers and to enter the subretinal space. Microglia gain access to RPE at the extracellular interface where photoreceptor outer-segment tips contact and closely oppose the RPE, leading to local proliferation, migration, enhanced phagocytosis, and secretion of cytokines and chemokines. The microglia-triggered inflammatory responses lead to photoreceptor and RPE cell death.^52,53^

### Exogenous *RS1* gene expression in *Rs1*-KO mouse retina suppresses immune system gene responses

We identified a major and rapid transition in the transcriptome landscape of *Rs1*-KO retina after exogenous *RS1* gene expression. Exogenous *RS1* attenuated the inflammatory gene expression that dominated the *Rs1*-KO diseased retina. Inflammatory gene expression profiles were reduced to low levels during functional recovery. In a process analogous to the Apoe/Trem2 “microglia switch,”^54^ *RS1* gene expression in *Rs1*-KO retina transitioned the inflammatory immune response to immune quiescence. Restoration of neural cell adhesion, improved structural integrity, and functional competence are important contributing factors in suppression of immune cell activation in the treated retina.

In retinal degenerations, reactive microglia in the outer retina exacerbate photoreceptor cell death.^50^ Microglial contribute to rod demise as demonstrated by live cell imaging in *Rd1* mouse with rapid photoreceptor degeneration. Studies in which retinal microglia were genetically ablated by administration of tamoxifen demonstrated that inhibition of microglial phagocytosis underlies the functional amelioration of photoreceptor degeneration.^55,56^ In retinal diseases, microglia modulation is being explored actively as a therapeutic strategy: therapies that modulate the general activation state of microglia in the disease context and those that modulate specific molecular pathways through which microglia exert pathological effects.^45,57,58^ While microglial inhibition is useful in limiting inflammatory responses, it would disrupt microglia homeostatic functions such as phagocytosis of retinal debris and other immune surveillance functions. Furthermore, there is evidence for constitutive, bidirectional communication between neurons and microglia in regulating neuronal activity and synaptic integrity and homeostasis in the retina and central nervous system.^59,60^ Developing immunomodulatory agents that stimulate microglia to adopt a regulatory phenotype without provoking cytotoxic effects is a major scientific and clinical challenge.

### RPE/photoreceptor signatures in *Rs1*-KO retina expressing exogenous *RS1* gene

In this transcriptome analysis, we wanted to identify gene expression networks that play a role in recovery and regeneration processes. Structural disorganization of the XLRS retina, rather than a deficiency of signaling molecules or phototransduction enzymes, causes vision loss in XLRS. Transcriptome profiles of *Rs1*-KO retina expressing exogenous *RS1* gene show distinct recovery patterns and gene signatures necessary to provide the structural framework toward a functional recovery. As photoreceptor cell death is an early event in XLRS mouse model,^53^ the response and recovery of photoreceptors after AAV8-*RS1* gene transfer are evident as early as 7 days postinjection. The transcriptome profile of *RS1*-KO retina expressing *RS1* gene is remarkably concordant with genes coding for wound healing, focal adhesion, anatomical structure formation, tissue development, camera-type eye development, and retinoid cycle enzymes (Fig. 3E). The upregulation of RPE cell-based retinoid biosynthesis genes linked to pathways for photopigment regeneration, and retinal/retinol transport carrier proteins (Rbp1 and Stra6), oxygen, and amino acid transporters are responses that replenish the reduced levels of rhodopsin and also sustain visual function under improved retinal homeostasis following *RS1* gene transfer. It is noteworthy that the differential gene expression profiles include a gene that determines the polarity of photoreceptor cells (secreted frizzled-related protein 1 [Sfrp1]).

Enzymes involved in melanin synthesis were also upregulated, that is, pigmentation enzymes Tyr, Dct, Tyrp1, and Oca2.^61,62^ Tyr is a key enzyme in pigment synthesis and is produced in RPE cells. Although the link between RS1 and Tyr appears unusual, Tyr was identified previously as a possible modifier of the XLRS phenotype.^63,64^ Johnson *et al.* postulated that l-dopa and dopamine products of tyrosine metabolism have a role in retinal development and function. Although our transcriptome analysis identified *Tyr*, the genetic modifier for XLRS, we do not understand how *Tyr* drives the phenotype variation. RPE signature genes become important because the progression of juvenile retinoschisis is associated with changes in RPE,^65^ secondary to the degenerative process in the adjacent neurosensory retina. Thus, these transcriptome profiles identify candidate genes that are crucial for enhancing structural and functional recovery of photoreceptors in XLRS retina. Given the intimate anatomic and functional relationship of RPE cells and photoreceptors in visual cycle, these transcript signatures reflect cell function-specific endpoints.

### *RS1*-induced gene expression reveals significant changes in basement membrane collagens

Transcriptome profiling of *Rs1*-KO retinas expressing exogenous *RS1* identified multiple collagen genes. Collagens are the most abundant proteins in ECM. During the early recovery phase, collagens implicated in vasculature were upregulated (Col 8a1 and Col8a2), but fibril-forming and fibril-associated and transmembrane collagens were downregulated. By day 35, network forming collagen 4 isomeric alpha chains, Col4a3, Col4a4, Col4a5, Coll4a6, and other basement membrane components, Lama 2, Lama 3, heparan sulfate proteoglycan 2 (HSPG2/perlecan), and Sparc were upregulated. Sprac appears to regulate cell growth through interactions with the ECM and cytokines. Each Col4 molecule is a heterotrimer composed of three alpha chains: a1.a1.a2(4); a3.a4.a5(4); and a5.a5.a6(4). Col 4 heterotrimers and laminins self-assemble into supramolecular networks with nidogen and perlecan acting as bridges. In retina, Bruch's Membrane of RPE is an important anatomical structure with two distinct basement membranes.^66^ The innermost layer is a basal lamina secreted by the RPE cells, and the outermost layer is a basal lamina secreted by the vascular endothelium of the choriocapillaris. The RPE-basal lamina contains Col 4a3, Col4a4, and Col4a5, like the basement membrane of kidney glomeruli, another organ with specialized filtration and transport functions.^67^ Bruch's membrane maintains integrity of the RPE, which separates the retina from the choriocapillaris. RPE supports photoreceptor health, and RPE disruption is often a disease manifestation. At the innermost side of the retina, the inner limiting membrane (ILM) is histologically a basement membrane of Muller cells and forms a physical separation that protects the retina from traction from the vitreous as the eye moves.

The Col 4a isomeric chains are components of all basement membranes. Basement membranes are specialized extracellular matrices that form cell scaffoldings.^68,69^ Col 4a3, Col 4a4, and Col 4a5 are present in both the ILM/NFL and the RPE basement membrane. In Alport syndrome mutations, Col3a3, Col4a4, and Col4a5 cause thinning of both the ILM/NFL and the RPE Bruch's basement membrane.^70^ It is known that Col 4a3, Cola4, Col a5, and Cola6 maintain the mechanical stability of mature basal lamina, including ILM at stages of increasing mechanical demands.^71^ Both basement membrane and RS1 are integral components of the ECM and provide essential physical scaffolding for the cellular constituents and initiate crucial biochemical and biomechanical forces across length scales in the retina required for morphogenesis and homeostasis. In this context, it is interesting to note that in XLRS, microcysts form between the GCL and ILM where there is no RS1. Perhaps, the ECM, which is anchored to bipolar cells through RS1 in INL, extends up to GCL and ILM to maintain the integrity of retinal tissues between GCL and ILM. RS1 is more important for the process of ECM stabilization. In summary, this transcriptome study identifies *RS1* gene-driven signatures involved in structural and functional recovery of XLRS retina and provides candidates for future investigation of molecular processes underlying RS1 function in retinal development.

### How does the immune quiescence phenotype, triggered by provision of exogenous *RS1*, help retinal function in the context of *Rs1*-KO (XLRS) gene therapy?

While investigations continue to focus on microglia as a potential cellular target for therapy to treat inherited retinal degenerative diseases, we show, for the first time to our knowledge, that restoration of functional *RS1* in mouse XLRS retina using AAV8-*RS1* restores immune homeostasis of retinal tissue and alleviates XLRS pathology. These results link the functional recovery in XLRS-*RS1* retina to suppression of dysregulated immune phenotype triggered by schisis injury to cell membranes. In this study, we delivered the *RS1* gene at P14, which is just before the onset of structural schisis, and 7 days later, by P21, we observed microglia quiescence that correlated with transgene *RS1* expression and schisis resolution. We believe that restoration of a homeostatic state by the therapeutic transgene likely is integral to functional recovery, even though the transgene is not directly linked to microglia function. As discussed earlier in the context of developing immunomodulatory agents that stimulate microglia to adopt a regulatory phenotype, future gene therapy approaches should seriously consider making immunomodulation as an important ally of “simple” gene therapy, to arrest immune-mediated tissue damage and bring out long-term therapeutic benefit.

Our results demonstrate that early *RS1* gene therapy treatment would be more effective by overriding schisis-induced microglia activation and photoreceptor degeneration. In situations where gene therapy is employed late in the degenerative process, the magnitude of photoreceptor cell death and morphological disruption of the retina may limit the efficacy of the therapy. Ocular gene therapy in animal models and human patients may show functional rescue even though it does not fully stop the degenerative process, as was noted in *RPE65* gene therapy clinical trials for Leber congenital amaurosis in which loss of photoreceptors continued despite functional recovery in treated areas.^72,73^ Hence, suppressing the microglial activation and the associated inflammatory cascade through early therapeutic intervention could yield far-reaching therapeutic benefit on morphological preservation apart from the resolution of structural schisis in the retina.

The retina of P18 mice roughly corresponds to a 2-year-old child, and all retinal layers are fully formed by P16–18, although photoreceptors fully mature between P21 and P30. In XLRS mice, the disease phenotype and microglial activation become evident around P16–P18. In humans, photoreceptors in the fovea, which subserves high acuity vision, reach functional maturity around 48 months of age. We postulate that ocular gene therapy for inherited retinal diseases likely becomes more effective when therapeutic intervention is conducted at earlier ages before the retinal degeneration becomes irreversible. Since the XLRS retina initially exhibits normal retinal lamination during postnatal development, this indicates a time window to administer gene therapy when the “microglia switch” can be implemented before major destructive degeneration occurs. While the therapeutic window of opportunity for treatment remains to be known, information might be gleaned by regular monitoring of retinal structure of young XLRS patients, possibly using OCT.^74^

## Authors' Contributions

P.A.S., C.V., and Y.Z.: planned and designed the experiments. C.V. performed RNA analysis and statistics; M.J.B. conducted the RNA-Seq experiments and most analyses, and assembled transcriptomes and bioinformatics; Y.Z. performed IHC analyses and subretinal injections; C.V; wrote the article; R.N.F. and P.A.S. edited the article; C.V. and P.A.S. intellectually contributed to the study.

## Supplementary Material

Supplemental data

Supplemental data

Supplemental data

Supplemental data

Supplemental data

Supplemental data

Supplemental data

Supplemental data

Supplemental data

Supplemental data

Supplemental data

Supplemental data
