# Supplementary material for: Genetic Rescue of X-Linked Retinoschisis Mouse (Rs1−/y) Retina Induces Quiescence of the Retinal Microglial Inflammatory State Following AAV8-RS1 Gene Transfer and Identifies Gene Networks Underlying Retinal Recovery
Source: Hum Gene Ther. 2021 Jul 16;32(13-14):667–81. doi: 10.1089/hum.2020.213 (PMC8312029; doi:10.1089/hum.2020.213)
Supplement: Supplemental data [file Supp_Table1.pdf]

Table S1. **The number of sequencing reads generated for each sample and their alignment statistics**

Sample	Annotation	% Annotation	Genome	% Genome	Total Depth
P12_WT_1	41,052,774	83.9	46,577,438	95.2	48,923,036
P12_WT_2	35,231,979	85.5	39,159,111	95.1	41,194,548
P12_WT_3	40,763,850	85.7	46,103,182	97.0	47,542,843
P12_KO_1	40,609,231	86.3	45,566,316	96.8	47,052,159
P12_KO_2	40,980,476	86.6	46,074,661	97.4	47,309,482
P12_KO_3	39,629,420	87.0	44,358,998	97.3	45,575,240
P21_WT_1	43,658,296	79.8	52,610,761	96.2	54,713,454
P21_WT_2	34,111,976	81.6	40,459,476	96.8	41,813,069
P21_WT_3	33,674,896	81.6	40,047,749	97.1	41,249,999
P21_KO_1	45,576,264	83.2	52,908,262	96.5	54,799,018
P21_KO_2	34,864,059	82.9	40,361,731	95.9	42,072,527
P21_KO_3	40,617,639	84.1	46,438,289	96.2	48,274,969
C7_1	25,687,006	81.6	28,690,871	91.1	31,491,157
C7_2	26,737,648	82.9	29,756,469	92.2	32,265,532
C7_3	25,840,002	83.3	28,272,959	91.2	31,011,982
C7_4	28,544,917	82.9	31,535,688	91.6	34,427,051
G7_1	25,389,232	83.6	28,225,146	92.9	30,382,944
G7_2	23,730,564	82.7	26,203,062	91.3	28,709,428
G7_3	27,096,965	84.7	29,643,550	92.7	31,987,932
G7_4	24,814,856	81.8	28,068,038	92.5	30,337,652
C35_1	24,674,114	78.5	30,594,114	97.4	31,412,500
C35_2	23,643,028	81.1	28,496,688	97.7	29,160,368
C35_3	22,979,669	78.4	28,592,320	97.6	29,305,747
G35_1	22,736,717	79.3	27,954,542	97.5	28,668,315
G35_2	22,250,476	80.5	26,949,058	97.5	27,631,376
G35_3	18,696,996	79.5	22,804,649	96.9	23,528,894
G35_4	21,597,645	79.5	26,426,333	97.2	27,181,468

The alignment statistics refer to the number and percentage of reads that align to the genome and to the annotation used in the analysis.
