# Supplementary material for: Genetic Rescue of X-Linked Retinoschisis Mouse (Rs1−/y) Retina Induces Quiescence of the Retinal Microglial Inflammatory State Following AAV8-RS1 Gene Transfer and Identifies Gene Networks Underlying Retinal Recovery
Source: Hum Gene Ther. 2021 Jul 16;32(13-14):667–81. doi: 10.1089/hum.2020.213 (PMC8312029; doi:10.1089/hum.2020.213)
Supplement: Supplemental data [file Supp_Fig1.pdf]

Figure S1. RNA sequence pipeline and principal-component analysis

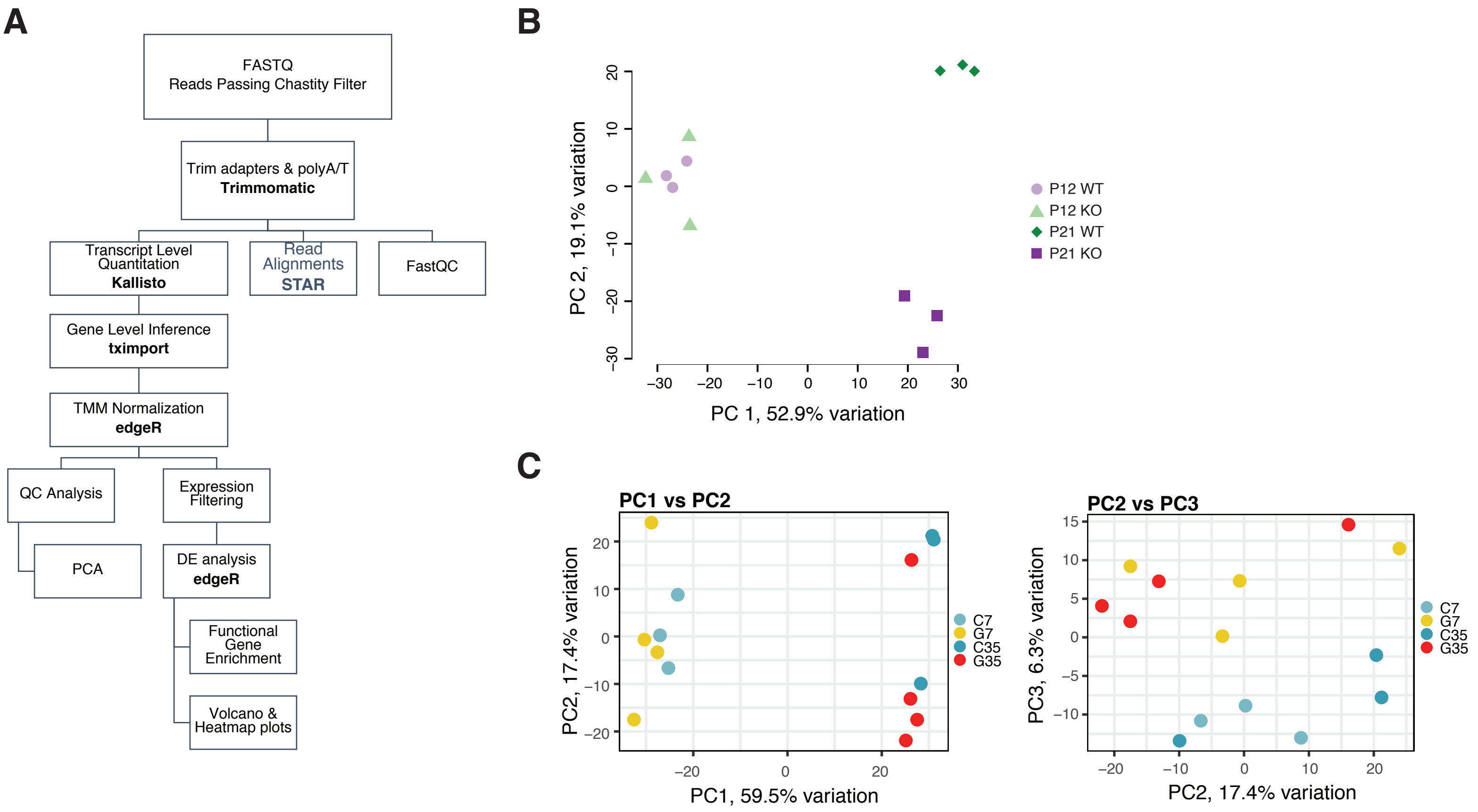

(A) Schematic of the RNA-seq analysis pipeline (B) Principal-component analysis (PCA) of the expressed CPM values for each sequencing sample P12 *Rs1*-KO vs WT. and P21 *Rs1*-KO vs. WT. Percentages indicate experimental variance assigned to each PC. (C) Principal-component analysis (PCA) of the expressed CPM values for each sequencing sample. G7 vs. C7 and G35 vs. C35. Percentages indicate experimental variance assigned to each PC.
