# Supplementary material for: Genetic Rescue of X-Linked Retinoschisis Mouse (Rs1−/y) Retina Induces Quiescence of the Retinal Microglial Inflammatory State Following AAV8-RS1 Gene Transfer and Identifies Gene Networks Underlying Retinal Recovery
Source: Hum Gene Ther. 2021 Jul 16;32(13-14):667–81. doi: 10.1089/hum.2020.213 (PMC8312029; doi:10.1089/hum.2020.213)
Supplement: Supplemental data [file Supp_Table2.pdf]

Table S2. List of TaqMan Primers, Antibodies and other reagents used in this study

Catalogue Number	Name	Source
Mm00437762_m1	B2m- beta-2 microglobulin	TFS [a]
Mm00504306_m1	Bcl3- B-cell CLL/lymphoma 3	TFS
Mm00437893_g1	C4b- complement component 4B (Chido blood group)	TFS
Mm00432142_m1	C1qa- complement C1q A chain	TFS
Mm00516817_m1	Cldn7- Claudin 7	TFS
Mm00432983_m1	Edn2- endothelin 2	TFS
Mm01285715_m1	Fgf2- fibroblast growth factor 2	TFS
Mm00516023_m1	Icam1-intercellular adhesion molecule 1	TFS
Mm01156845_m1	Ppef1-protein phosphatase with EF-hand domain 1	TFS
Mm00488076_m1	Rs1- retinoschisin 1 (mouse)	TFS
Mm00465263_m1	Zan1- zonadhesin	TFS
Mm01219775_m1	Stat3- signal transducer and activator of transcription3	TFS
Mm04277571_s1	18S ribosomal RNA	TFS
Hs99999901_s1	18s- human18S rRNA	TFS
Hs00171245_m1	RS1-retinoschisin 1 (human)	TFS
304437	TaqMan™ Universal PCR Master Mix	TFS

Antibodies and other reagents

Catalogue number	Name	Source
MCA 1957	Rat anti Mouse CD68 antibody, clone FA-11. 1:400	Bio-Rad [b]
019-19741	Guinea Pig Anti-IBA1 Synaptic Systems. 1:500	Synaptic Systems [c]
Custom made	Rabbit Anti-Retinoschisin. 1:1000	TFS
A-11036	Goat anti-Rabbit IgG (H+L) Secondary Antibody, Alexa Fluor 568. 1:1000	TFS
A-11073	Goat anti-Guinea Pig IgG (H+L), Secondary Antibody, Alexa Fluor 488. 1:1000	TFS
A-21208	Donkey anti-Rat IgG (H+L), Secondary Antibody, Alexa Fluor 488. 1:1000	TFS
D9542	DAPI-4′,6-Diamidino-2-phenylindole dihydrochloride. 1: 5000	Sigma-Aldrich[d]

[a]Thermo Fisher Scientific, 81 Wyman St, Waltham, MA 02145 USA; [b]Bio-Rad, 2000 Alfred Nobel Drive Hercules, California 94547; Synaptic Systems, Sigma-Aldrich, PO Box 14508. St. Louis, MO 63178. USA.
