# Supplementary material for: Genetic Rescue of X-Linked Retinoschisis Mouse (Rs1−/y) Retina Induces Quiescence of the Retinal Microglial Inflammatory State Following AAV8-RS1 Gene Transfer and Identifies Gene Networks Underlying Retinal Recovery
Source: Hum Gene Ther. 2021 Jul 16;32(13-14):667–81. doi: 10.1089/hum.2020.213 (PMC8312029; doi:10.1089/hum.2020.213)
Supplement: Supplemental data [file Supp_Table3.pdf]

Table S3. Significantly Differentially Expressed Gene (DEGs) List. *Rs1*-KO vs. WT at P21

Gene	Transcript Count	Log FC	Ave. Expression	P. Value	adj. P. Val	B
Rs1	3	-5.36459	8.54	1.44E-12	2.11E-08	18.65226
Ppef1	3	5.562406	4.03	1.06E-10	5.19E-07	13.38634
Edn2	1	5.322363	3.41	8.40E-11	5.19E-07	13.07035
C4b	3	4.979958	4.62	7.95E-10	2.90E-06	12.69865
Sepp1	7	2.268762	5.91	4.04E-09	7.78E-06	11.82036
Lrrc2	3	2.1965	6.19	4.26E-09	7.78E-06	11.77467
Fgf2os	1	3.86026	3.61	2.90E-09	7.06E-06	11.52813
E2f6	1	1.539739	5.28	6.80E-09	1.10E-05	11.30202
C1qa	1	2.619059	3.61	8.00E-09	1.17E-05	10.87511
Lad1	3	4.388053	2.37	2.55E-09	7.06E-06	10.8401
Rnf144b	2	1.313213	5.82	1.19E-08	1.58E-05	10.75171
B2m	1	2.141272	4.97	1.85E-08	2.08E-05	10.30848
C1qb	1	2.754169	3.81	2.11E-08	2.20E-05	10.05993
H2-D1	4	1.517083	5.39	2.94E-08	2.53E-05	9.844193
Trem2	4	3.302019	2.43	1.81E-08	2.08E-05	9.75111
Mt2	1	2.898466	4.66	3.28E-08	2.63E-05	9.729906
Csf1r	2	2.342391	4.83	3.49E-08	2.63E-05	9.68004
Gm15983	1	1.629593	5.52	3.83E-08	2.63E-05	9.572016
H2-K1	9	1.933763	4.53	3.98E-08	2.63E-05	9.548351
Fcgr3	4	3.091408	2.3	2.46E-08	2.39E-05	9.492512
Mpeg1	1	3.694453	4.15	4.21E-08	2.63E-05	9.426489
Gfap	3	2.543331	5.71	4.47E-08	2.63E-05	9.419098
Gnb3	4	1.468042	8.86	4.33E-08	2.63E-05	9.351035
Klhl29	1	1.400407	6.02	4.78E-08	2.63E-05	9.319867
Fcrls	2	3.680731	2.14	2.80E-08	2.53E-05	9.257703
Cx3cr1	2	2.213516	3.5	5.30E-08	2.67E-05	9.198517
Sec22c	5	1.191877	7.67	5.52E-08	2.69E-05	9.114187
C1qc	2	2.90504	3.87	6.05E-08	2.85E-05	9.090567
Ctss	2	2.197745	4.65	6.88E-08	3.14E-05	9.006774
Jak3	5	2.851394	2.07	4.76E-08	2.63E-05	8.915463
Antxr2	3	2.685368	4.51	8.31E-08	3.58E-05	8.818659
Pcolce	12	2.325422	4.95	8.34E-08	3.58E-05	8.808967
Cd53	1	2.862634	1.29	5.01E-08	2.63E-05	8.56565
Tnnt2	15	3.740512	2.64	9.98E-08	3.98E-05	8.395787
Fgf2	8	3.142569	6.53	1.17E-07	4.27E-05	8.392376
H2-Q4	2	1.803411	3.23	1.30E-07	4.52E-05	8.342342
Pdzph1	3	-1.04808	8.26	1.15E-07	4.27E-05	8.318886
Bst2	1	2.588175	1.55	8.88E-08	3.71E-05	8.26976
Laptm5	10	2.739791	3.07	1.37E-07	4.53E-05	8.253014
Glb1l3	1	-2.98668	2.21	1.36E-07	4.53E-05	8.109352

Kremen1	2	1.240757	5.76	2.18E-07	6.92E-05	7.740565
Cebpd	1	2.776443	3.86	2.61E-07	7.83E-05	7.682142
Gadd45b	1	2.046828	3	2.79E-07	7.98E-05	7.5961
Tyrobp	3	3.020541	2.44	2.60E-07	7.83E-05	7.563688
Myo1f	4	3.548627	0.56	1.21E-07	4.31E-05	7.53996
Hexb	1	1.416211	5.56	2.87E-07	8.08E-05	7.466647
Mt1	1	1.769827	5.47	3.13E-07	8.46E-05	7.392133
Slc6a2	2	3.209548	2.06	3.18E-07	8.46E-05	7.308293
Agtpbp1	22	1.284392	8.6	3.08E-07	8.46E-05	7.259721
Lgals3bp	5	1.483505	4.01	4.00E-07	9.37E-05	7.243112
Gm10030	3	1.499563	3.85	4.04E-07	9.37E-05	7.240182
Myo10	13	1.193445	6.96	3.28E-07	8.56E-05	7.236936
Gm16867	7	1.255596	4.67	4.15E-07	9.49E-05	7.152438
Agrn	7	1.222994	7.06	3.57E-07	8.77E-05	7.143232
Trf	9	2.064224	7.21	3.60E-07	8.77E-05	7.134784
A2m	3	3.534161	4.75	4.55E-07	0.000102	7.112246
Rabgef1	9	-1.12809	8.23	3.60E-07	8.77E-05	7.096322
Serpina3n	1	4.227071	4.38	4.86E-07	0.000103	7.065114
Prtg	2	1.710948	4.17	5.09E-07	0.000105	6.990273
Apobec1	9	2.726759	1.65	5.10E-07	0.000105	6.843387
Fhad1	8	1.787019	2.29	6.02E-07	0.000117	6.8134
Bcl3	6	5.05498	1	3.53E-07	8.77E-05	6.770225
Fcgr2b	7	3.53592	1.37	5.58E-07	0.000111	6.656808
Cfi	2	2.07837	1.54	6.36E-07	0.000119	6.653582
Tgm2	4	1.579609	3.26	7.81E-07	0.000137	6.595753
Vcam1	4	1.168031	4.39	7.32E-07	0.000135	6.585504
Wdfy4	4	3.976334	-0.31	2.72E-07	7.96E-05	6.541561
Adgre1	2	3.68174	1.29	6.23E-07	0.000119	6.538922
Stat3	14	1.617044	7.07	6.35E-07	0.000119	6.525608
Unc93b1	4	2.406691	2.55	8.52E-07	0.000148	6.493895
Fn1	17	1.031186	6.2	7.52E-07	0.000136	6.384298
Junb	1	1.332954	4.61	8.88E-07	0.000149	6.36417
Cyth4	1	2.183564	1.51	8.90E-07	0.000149	6.348637
2810032G03Rik	2	2.018415	1.88	9.49E-07	0.000152	6.342499
Ly6e	16	1.0764	5.1	9.21E-07	0.000151	6.265989
Ly86	1	1.997778	2.47	1.08E-06	0.000163	6.264408
Gpr34	2	2.180863	1.45	9.78E-07	0.000155	6.255348
Mlf1	4	1.156957	3.77	1.14E-06	0.000168	6.179653
Il10ra	3	2.293988	0.89	9.92E-07	0.000156	6.135389
Lyz2	1	4.883875	3.19	1.43E-06	0.000191	5.98976
Osmr	6	1.836776	3.88	1.39E-06	0.000188	5.982328
Gm12802	1	2.927223	3.13	1.49E-06	0.000195	5.9576
Pbxip1	5	1.236919	5.93	1.17E-06	0.00017	5.938087
Myo5b	11	1.132517	5.47	1.22E-06	0.000174	5.929234

C1ra	1	2.010114	1.63	1.49E-06	0.000195	5.899575
Itgam	6	1.629523	2.81	1.61E-06	0.000203	5.880033
Btc	4	1.905675	3.93	1.58E-06	0.000201	5.846078
Syt15	3	1.18492	3.93	1.69E-06	0.000207	5.761066
Rbm17	6	1.034837	6.21	1.37E-06	0.000188	5.74087
Slc1a1	5	1.021501	5.91	1.45E-06	0.000193	5.697615
Pdlim3	1	1.069679	4.2	1.77E-06	0.000213	5.68082
Il17ra	3	1.349266	2.45	2.08E-06	0.000237	5.631687
Socs3	3	2.240149	2.74	2.10E-06	0.000237	5.622132
Pld4	1	2.794211	1.75	2.14E-06	0.000237	5.558369
Gm37904	1	2.91267	1.08	1.98E-06	0.00023	5.545098
Rnase4	2	2.042237	2.55	2.28E-06	0.000243	5.541364
Wwtr1	2	1.158731	4.79	1.91E-06	0.000223	5.527664
Calca	7	2.442037	0.95	2.07E-06	0.000237	5.504937
Tnfrsf1a	6	1.635929	3.57	2.27E-06	0.000243	5.49284
Fyb	8	2.744067	0.73	2.14E-06	0.000237	5.423636
Ms4a7	5	1.912568	0.97	2.38E-06	0.000249	5.396562
C3ar1	1	4.68256	0.58	1.89E-06	0.000223	5.381981
Gbp3	7	2.243825	1.92	2.66E-06	0.000266	5.374319
Atf3	3	1.785837	3	2.68E-06	0.000266	5.365132
Ttc39aos1	2	1.326864	2.71	2.95E-06	0.000285	5.277082
Slc25a37	4	1.52725	6.87	2.13E-06	0.000237	5.228926
Bcl6	2	1.144998	4.85	2.58E-06	0.000262	5.19822
Hmha1	5	1.843046	1.43	3.36E-06	0.000307	5.129135
Fxyd3	11	3.097417	0.73	3.04E-06	0.000293	5.105412
Timp2	2	1.006496	6.54	2.41E-06	0.00025	5.102793
Ncf1	7	1.546818	2.07	3.55E-06	0.000314	5.101715
Cd47	2	1.059343	6.47	2.76E-06	0.000272	4.964519
Clec7a	4	4.324545	0.56	3.52E-06	0.000314	4.890274
Zbtb7c	2	1.824776	1.85	4.61E-06	0.000378	4.844927
Nckap1l	1	1.692187	2.8	4.58E-06	0.000378	4.829354
Vav1	5	2.963201	0.21	3.82E-06	0.000329	4.82351
Zan	5	-1.27666	5.98	3.29E-06	0.000303	4.804569
Lgi4	4	1.479873	3.1	4.80E-06	0.000388	4.758164
RP23-100P8.3	1	-1.41143	3.8	4.56E-06	0.000378	4.729204
Itgb2	5	2.709608	0.93	5.07E-06	0.000407	4.689568
Flnc	3	2.405153	1.27	5.28E-06	0.000415	4.684952
Csf2rb	1	2.757663	-0.045	4.63E-06	0.000378	4.620928
Spidr	1	1.131897	3.09	5.51E-06	0.000428	4.611996
Cd180	5	2.817143	0.45	5.16E-06	0.00041	4.609068
RP23-457J22.1	1	7.033131	-1.66	1.12E-06	0.000168	4.602841
Aox1	2	1.447799	1.88	6.81E-06	0.00049	4.462236
Kctd12	2	1.232904	3.47	6.15E-06	0.000456	4.459951
AF251705	2	2.781759	0.15	6.07E-06	0.000455	4.423676

Xlr3b	3	1.512504	2.28	7.22E-06	0.00051	4.394108
2810030D12Rik	4	-1.14548	3.71	6.35E-06	0.000468	4.388411
Micall2	4	1.414021	1.55	7.50E-06	0.000519	4.367792
Skap2	7	1.404087	2.35	7.46E-06	0.000519	4.358093
Rfx2	2	1.800754	4.25	6.42E-06	0.000471	4.334246
Psmb8	3	1.692194	1.72	7.88E-06	0.000532	4.319305
Gm14091	1	-2.26361	1.74	7.90E-06	0.000532	4.317259
BC026585	5	1.179213	3.43	7.21E-06	0.00051	4.297107
Il6ra	2	2.01351	1.36	8.21E-06	0.000548	4.273836
Tgm7	1	-1.86045	0.27	7.62E-06	0.000523	4.270658
Gm11754	1	-2.11861	0.84	8.25E-06	0.000548	4.246366
Tmlhe	1	-1.12345	5.18	5.95E-06	0.000448	4.24559
Rgs10	6	1.215647	2.7	8.59E-06	0.000568	4.189709
Syk	5	2.21487	0.76	9.31E-06	0.000607	4.12288
Ttll3	9	-1.03394	4.97	7.05E-06	0.000505	4.08923
Hpgds	3	2.266183	1.76	1.04E-05	0.000656	4.050141
Sla	5	2.768025	0.07	9.44E-06	0.00061	4.02524
Icam1	1	1.985802	0.79	1.09E-05	0.000673	3.981939
Msn	3	1.021714	5.27	7.61E-06	0.000523	3.977009
Ifitm3	1	1.648039	3.41	1.01E-05	0.000645	3.961075
Ms4a6c	2	3.463953	0.12	1.03E-05	0.000652	3.936923
Fcer1g	3	2.166495	2.07	1.27E-05	0.000747	3.837794
Rab7b	5	2.035995	0.66	1.28E-05	0.000747	3.822204
Cd68	3	3.170169	1.94	1.32E-05	0.00076	3.816364
Serping1	3	2.172205	4.42	1.12E-05	0.000682	3.738589
Cebpa	2	2.123857	0.68	1.46E-05	0.000824	3.697302
Slco2b1	9	1.087859	3.03	1.38E-05	0.000787	3.668562
Fcgr1	2	3.161248	0.4	1.62E-05	0.000871	3.566252
Lin7b	1	1.298025	2.51	1.66E-05	0.00089	3.527266
Il10rb	5	1.187569	2.32	1.73E-05	0.000913	3.501536
Cd33	6	2.798936	1.54	1.84E-05	0.000952	3.487604
Cd52	3	2.505228	0.16	1.76E-05	0.000921	3.4803
P2ry13	1	1.780057	0.86	1.92E-05	0.000972	3.44648
Trim30a	4	1.778862	1.17	2.01E-05	0.000995	3.404709
Tmem176a	7	1.101178	4.14	1.55E-05	0.000851	3.387508
Hvcn1	5	1.48037	1.18	2.10E-05	0.001011	3.362535
Aif1	7	2.334349	0.53	2.07E-05	0.001011	3.358852
Tril	1	1.141345	3.81	1.70E-05	0.000901	3.338676
Arhgap30	4	2.006999	0.46	2.15E-05	0.001028	3.326177
Knstrn	9	-1.96794	2.4	2.08E-05	0.001011	3.313805
Scube1	5	2.674894	1.86	2.18E-05	0.00103	3.313545
Myof	2	1.170702	4.45	1.61E-05	0.000871	3.290614
Gpnmb	5	2.937172	4.76	1.69E-05	0.000898	3.286783
Bag3	1	1.232968	2.18	2.18E-05	0.00103	3.279563

Selenbp1	5	-1.58386	5.12	1.51E-05	0.000843	3.259647
Il13ra1	1	1.378434	0.91	2.37E-05	0.001084	3.241686
Hsd17b2	1	2.102877	0.89	2.42E-05	0.00109	3.223323
Dab2	14	1.991499	1.66	2.43E-05	0.001093	3.2054
Clec2e	1	-1.27172	2.74	2.36E-05	0.001084	3.13368
Ucp2	11	1.011692	4.4	1.89E-05	0.000964	3.124661
Wipf1	7	1.213345	2.12	2.61E-05	0.001142	3.098011
Blnk	3	1.641797	1.1	2.80E-05	0.001187	3.078686
Clec2d	1	1.091448	1.95	2.73E-05	0.001168	3.062586
Gm765	2	-1.54	0.73	3.08E-05	0.001241	2.987
Cyba	1	1.442734	1.74	3.04E-05	0.001236	2.973136
Rad51	8	-1.01603	2.4	2.88E-05	0.001203	2.963737
Nfkbie	1	1.371529	1.21	3.21E-05	0.001272	2.940096
Oasl2	9	1.6655	3.67	2.60E-05	0.001142	2.9252
Loxl4	5	1.634247	3.28	2.76E-05	0.001175	2.918423
Vsir	7	1.763949	2.6	3.00E-05	0.001224	2.917126
Il1r1	3	1.135525	2.99	2.92E-05	0.001209	2.885098
Ctsz	1	1.148106	4.8	2.52E-05	0.001118	2.757761
Ikzf1	6	1.989688	0.21	3.87E-05	0.001431	2.756687
Spata6	14	1.174192	3.28	3.19E-05	0.001268	2.751185
Fgd2	8	2.205665	0.47	4.07E-05	0.001477	2.714054
Cybb	5	2.81731	0.59	4.20E-05	0.001492	2.685545
Map3k8	5	1.377276	1.53	4.10E-05	0.001481	2.683539
D8Ertd82e	3	1.594599	1.52	4.16E-05	0.001488	2.668153
Trim30d	3	2.338382	0.69	4.31E-05	0.001508	2.660562
Ighm	2	1.890531	0.41	4.48E-05	0.001541	2.62337
AU020206	2	1.622382	1.37	4.62E-05	0.001571	2.573502
Steap4	1	3.108007	0.96	4.76E-05	0.001597	2.564464
Irf5	6	1.574843	1.04	4.76E-05	0.001597	2.554996
Rpl36-ps9	1	-1.52864	1	4.82E-05	0.001599	2.544108
Bin2	7	2.251222	0.26	4.99E-05	0.001633	2.516833
Hpdl	1	-1.4903	1.39	5.22E-05	0.001691	2.444196
Pik3cd	14	1.013509	2.77	4.62E-05	0.001571	2.430706
Gbp9	4	1.566049	1.79	5.28E-05	0.001697	2.408589
Gpc3	2	1.012263	3.45	4.37E-05	0.001514	2.388998
Wnt9b	1	-1.80409	1.68	5.44E-05	0.001727	2.385086
Nlrc5	6	2.830404	0.23	5.82E-05	0.001806	2.36635
Irf8	7	1.783125	1.51	5.63E-05	0.001763	2.365604
Tgfbr2	2	1.32892	2.86	5.34E-05	0.00171	2.274056
Plpp2	4	-1.16454	5.59	3.51E-05	0.001342	2.273213
Timp1	2	2.200556	0.55	6.45E-05	0.001905	2.269659
Nudt6	10	1.210648	1.98	6.08E-05	0.001867	2.242408
Ptpn6	11	1.82806	1.18	6.56E-05	0.001924	2.232669
Evi2a	5	2.099954	0.22	6.71E-05	0.001951	2.232077

Muc2	8	1.060897	2.14	6.08E-05	0.001867	2.223778
C3	4	2.405308	1.8	6.45E-05	0.001905	2.215337
Tmem71	1	1.934295	0.29	6.88E-05	0.001958	2.20853
Slc3a1	1	-1.09568	1.44	6.59E-05	0.001924	2.202175
Gatm	3	1.35381	1.22	6.76E-05	0.001952	2.195862
H2-T23	4	1.204681	2.58	5.96E-05	0.00184	2.194062
Grn	10	1.388875	5.62	3.93E-05	0.00144	2.164756
Tlr2	1	2.153766	0.48	7.22E-05	0.001999	2.160763
Zc3hav1	4	1.691958	1.71	6.80E-05	0.001955	2.158762
F630040K05Rik	2	-1.56265	5.36	4.16E-05	0.001488	2.128189
Klhl6	4	1.845166	0.38	7.56E-05	0.002037	2.117231
Mrc1	2	3.067374	1	7.53E-05	0.002037	2.112534
Hpse	2	1.369527	1.74	7.24E-05	0.001999	2.087795
Itgb8	3	1.130772	4.71	5.06E-05	0.001652	2.01943
Tagln2	4	1.613056	3.05	6.74E-05	0.001951	2.005869
Ptprc	13	1.767865	1.52	8.33E-05	0.002192	1.968563
Plxnd1	5	1.103044	3.44	6.60E-05	0.001924	1.954185
Rtp3	3	1.318914	1.24	8.84E-05	0.002258	1.923053
Phlda3	2	1.116912	2.18	8.17E-05	0.002158	1.91355
Gm10857	2	-2.36785	0.02	9.77E-05	0.002379	1.870407
3010001F23Rik	5	-1.48393	3.5	7.11E-05	0.00199	1.865621
Col24a1	4	1.110159	0.93	9.55E-05	0.002345	1.863491
Ltbr	3	1.129217	3.14	7.54E-05	0.002037	1.861392
Tmem98	1	1.131313	3.54	7.29E-05	0.002004	1.831324
Scn4a	2	-1.04513	4.9	5.75E-05	0.001796	1.829236
H2-Q7	6	1.123279	2.23	8.98E-05	0.002266	1.808722
C130080G10Rik	5	-1.41735	2.03	9.28E-05	0.00231	1.796788
Tmem26	1	1.249696	3.56	7.73E-05	0.002076	1.769434
A530058N18Rik	2	-1.15651	1.72	0.0001	0.002409	1.748306
Ptgs1	5	1.537662	1.26	0.000106	0.002513	1.738036
Lgals9	4	1.430116	2.14	9.83E-05	0.00239	1.73153
Parp14	3	1.600255	3.01	8.81E-05	0.002258	1.728073
Ugt1a6a	4	1.926553	0.67	0.000114	0.002615	1.702313
Dock2	7	2.14893	1.1	0.000112	0.002587	1.700325
Fcamr	2	1.328935	1.39	0.00011	0.002563	1.687161
Ppargc1b	3	-1.03579	6.24	5.81E-05	0.001806	1.658651
Slc38a5	4	1.28096	0.71	0.00012	0.002693	1.648458
Hdc	5	-2.43056	1.22	0.000118	0.002654	1.644806
Rab32	1	1.283058	0.64	0.000129	0.002788	1.580356
Dusp18	2	1.016886	1.31	0.000126	0.002765	1.551177
Ptgfr	3	2.219725	0.59	0.000136	0.002872	1.532802
Marveld3	3	1.107676	3.34	9.96E-05	0.002406	1.531946
Abcc3	6	2.533197	0.09	0.000141	0.002941	1.513493
Crym	2	1.514085	6.23	7.06E-05	0.001986	1.465167

2310030A07Rik	1	-1.11163	2.48	0.000122	0.002714	1.445744
Plcg2	1	1.286844	1.18	0.000143	0.002943	1.441539
Mef2b	4	-1.33051	0.83	0.000153	0.003049	1.397958
Gm16055	1	-1.92209	3.46	0.000112	0.002587	1.395112
Prph	2	1.291532	2.8	0.000131	0.002804	1.334733
Sh2d1a	7	-1.20024	2.35	0.000143	0.002951	1.298921
Slc37a2	3	1.120067	1.62	0.00016	0.003142	1.28053
Gm8995	2	1.887127	0.77	0.000175	0.00331	1.273796
Zfp92	2	1.077455	1.43	0.000172	0.003279	1.225697
Plin2	8	1.081837	3.02	0.000142	0.002941	1.20943
Tlr7	6	3.563313	0.99	0.000192	0.003489	1.188326
Col17a1	4	1.161906	3.26	0.000142	0.002941	1.16848
1600014C23Rik	1	-1.11292	0.75	0.000194	0.003521	1.158706
Adgrg6	4	1.144142	2.34	0.00017	0.003256	1.125852
Insl5	2	-1.12156	2.17	0.000177	0.003332	1.105728
Tmem173	1	1.317155	0.43	0.00021	0.003666	1.105632
Fosb	7	1.698863	2.53	0.000177	0.003332	1.068168
Esrp1	7	-1.42145	0.71	0.000214	0.003679	1.066659
S1pr2	1	1.176934	1.4	0.000204	0.003627	1.053393
Siglec1	3	1.419773	0.63	0.000219	0.003726	1.050895
Tmem132b	3	1.062464	4.73	0.000125	0.002763	1.031017
4833428L15Rik	1	-1.40401	0.74	0.000224	0.003771	1.016571
Inpp5d	11	1.281788	2.43	0.000191	0.003477	0.996163
Cd44	9	1.404865	4.06	0.000147	0.002963	0.994327
Gbp2	2	2.133064	2.05	0.000217	0.003702	0.932984
Gna14	1	1.162536	1.84	0.000219	0.003726	0.929691
Pdlim4	5	1.001569	3.14	0.000182	0.003384	0.922835
Rac2	1	1.604035	1.46	0.000235	0.003883	0.910365
Cldn7	5	-1.99079	0.81	0.000255	0.004041	0.888693
Glipr2	2	1.426641	0.64	0.00026	0.004088	0.877924
Kif4	3	-1.14011	1.84	0.00023	0.003831	0.872727
1700119H24Rik	1	-1.23239	1.54	0.000243	0.003957	0.856228
Dysf	16	1.080152	1.2	0.000257	0.004067	0.836206
Fabp4	2	-1.12245	2.57	0.000218	0.003715	0.821714
Lif	3	2.562609	0.39	0.000283	0.004279	0.820288
P2ry12	5	1.033961	1.98	0.000247	0.00398	0.786379
Kremen2	1	-1.11525	1.35	0.000269	0.004152	0.773072
Arpc1b	11	1.215679	3.24	0.000208	0.003652	0.76913
Naprt	1	1.065871	1.17	0.000278	0.004242	0.759491
Gm18289	1	-1.17738	0.65	0.000292	0.004357	0.756775
Pik3cg	4	1.293896	1.1	0.000286	0.004295	0.740534
A930031H19Rik	2	-1.7314	1.81	0.000266	0.004126	0.73556
BC028471	6	-1.1992	1.1	0.000294	0.004372	0.70699
H2-M3	1	1.39409	0.51	0.000316	0.004588	0.693033

Apoe	11	1.019721	9.73	0.000127	0.002769	0.678935
Mcm10	10	-1.23897	1.94	0.000283	0.004279	0.644301
Tmprss7	5	-1.17958	1.22	0.000335	0.004749	0.561033
C1s1	5	1.186432	1.33	0.000334	0.004749	0.553899
Cd109	2	1.588996	-0.86	0.00041	0.005247	0.497365
Fblim1	15	1.221367	0.89	0.000394	0.005131	0.434514
Synpo	12	1.12725	2.6	0.000318	0.004609	0.427656
Cd74	3	2.993108	0.66	0.00042	0.0053	0.417524
Lyn	4	1.158071	2.54	0.000326	0.004673	0.41198
Parp9	7	1.045298	2.79	0.000317	0.004601	0.397864
Tifab	1	1.254797	0.73	0.000424	0.005327	0.375601
Arhgap22	8	1.062544	1.09	0.000415	0.005264	0.358643
Asf1b	3	1.098979	1.59	0.000392	0.005115	0.356894
Pik3ap1	1	1.262216	1.41	0.000413	0.005256	0.328038
Gm11961	2	-1.05947	2.96	0.000328	0.004688	0.32505
Gm8251	1	-1.01568	1.17	0.000427	0.005351	0.314686
Rtkn2	6	1.255934	1.69	0.000425	0.005337	0.263621
Lcp1	11	1.110987	3.21	0.000338	0.004773	0.255664
Tlr4	4	1.357094	0.47	0.000492	0.005908	0.248798
Tectb	6	1.718207	0.89	0.000478	0.005793	0.243673
Adamtsl5	7	1.048035	2.92	0.00036	0.004894	0.239683
Stab1	13	1.369317	3.71	0.000325	0.004672	0.208895
Gbp7	2	1.247821	2.81	0.000379	0.005017	0.208498
Prelp	2	1.124683	5.86	0.000239	0.003922	0.169169
RP23-449M8.5	2	-1.30443	2.36	0.000433	0.005395	0.137851
C4bp-ps1	1	-1.55056	0.93	0.00053	0.006149	0.126005
Slfn5	4	1.109433	2.03	0.000475	0.00577	0.096157
Lpin3	5	1.669934	0.7	0.000574	0.006507	0.075644
Gbp6	3	1.330741	3.22	0.000406	0.00522	0.063375
H19	5	1.757524	0.83	0.000599	0.006678	0.019778
Abca9	2	1.105119	2.89	0.00045	0.005537	0.009874
Tmco4	4	1.131509	0.95	0.000604	0.006712	-0.01022
Nfatc1	4	1.48133	0.59	0.000649	0.007066	-0.04103
Cd22	15	1.073071	0.83	0.000652	0.007098	-0.07568
Spp1	7	2.194994	4.95	0.000378	0.005007	-0.13844
Kdelr3	1	1.120307	1	0.000724	0.007578	-0.20239
Cyr61	1	1.112261	1.99	0.000644	0.007032	-0.21643
Pros1	3	1.07825	3.96	0.000462	0.005653	-0.22643
Nfkb2	2	1.281216	1.73	0.000681	0.007292	-0.23344
Rnf183	3	-1.19479	0.67	0.000796	0.007995	-0.26546
Gm8956	1	-1.00927	1.84	0.000712	0.007527	-0.30706
Irgm2	3	1.340165	1.89	0.000738	0.00767	-0.33759
Fli1	2	1.050789	1.03	0.00085	0.008377	-0.37109
Ifih1	5	1.256599	2.51	0.000727	0.00759	-0.42695

Pycard	2	1.726713	1.06	0.000929	0.008776	-0.45549
Ncf2	6	1.02667	2.26	0.00078	0.007907	-0.46194
Gm12996	1	-1.8641	0.41	0.00107	0.009613	-0.53103
Cmtm3	1	1.001929	1.27	0.000977	0.009095	-0.54574
Gm29427	1	-1.03385	3.22	0.00074	0.007678	-0.59118
Kcng1	3	1.005854	1.29	0.001097	0.009775	-0.6685
Bub1b	4	1.248703	4.8	0.000687	0.007337	-0.81123
Gm20425	1	1.150972	3.21	0.000929	0.008776	-0.81697
Crxos	6	-1.06494	4.57	0.000757	0.007759	-0.89338
Rn18s-rs5	2	-1.20762	9.17	0.00101	0.009262	-1.56074
