# Supplementary material for: Genetic Rescue of X-Linked Retinoschisis Mouse (Rs1−/y) Retina Induces Quiescence of the Retinal Microglial Inflammatory State Following AAV8-RS1 Gene Transfer and Identifies Gene Networks Underlying Retinal Recovery
Source: Hum Gene Ther. 2021 Jul 16;32(13-14):667–81. doi: 10.1089/hum.2020.213 (PMC8312029; doi:10.1089/hum.2020.213)
Supplement: Supplemental data [file Supp_Table4.pdf]

Table S4. Significant Enriched GO Terms of DEGs in *Rs1*-KO retina at P21-Biological Processes

(A-Annotated Count; S- Significant Count; FDR-False Detection Rate)

2 **GO:0002376 immune system process** A1311/ S124 FDR 8.19E-28

A2m,Adgre1,Aif1,Apobec1,B2m,Bcl3,Bcl6,Blnk,Bst2,C1qa,C1qb,C1qc,C1ra,C1s1,C3,C3ar1,C4b,Cd109,Cd180,Cd44,Cd47,Cd74,Cebpa,Cebpd,Cfi,Clec2d,Cmtm3,Col24a1,Csf1r,Ctss,Cx3cr1,Cyba,Cybb,Dab2,Dock2,Edn2,Fcamr,Fcer1g,Fcgr1,Fcgr2b,Fcgr3,Fli1,Fyb,Gbp2,Gbp3,Gbp6,Gbp7,Gbp9,Gpc3,H2-D1,H2-K1,H2-M3,H2-Q4,H2-Q7,H2-T23,Icam1,Ifih1,Ifitm3,Ighm,Ikzf1,Il10rb,Il17ra,Il1r1,Inpp5d,Irf5,Irf8,Itgam,Itgb2,Jak3,Junb,Klhl6,Lcp1,Lgals9,Lif,Ltbr,Ly86,Lyn,Map3k8,Mlf1,Mrc1,Msn,Myo1f,Ncf1,Nckap1l,Nfatc1,Nfkb2,Nlrc5,Oasl2,P2ry12,Parp9,Pik3ap1,Pik3cd,Pik3cg,Plcg2,Pld4,Psmb8,Ptpn6,Ptprc,Pycard,Rab32,Rab7b,Rac2,Rtkn2,Serping1,Skap2,Spp1,Syk,Tgfbr2,Tlr2,Tlr4,Tlr7,Tmem173,Tmem176a,Tnfrsf1a,Trem2,Trf,Tril,Trim30a,Tyrobp,Unc93b1,Vav1,Vcam1,Vsir,Zc3hav1

7 GO:0002252 immune effector process A 420/ S59 FDR 8.19E-28

A2m,Apobec1,B2m,Bcl3,Bcl6,Bst2,C1qa,C1qb,C1qc,C1ra,C1s1,C3,C4b,Cd180,Cd47,Cd74,Cfi,Clec2d,Cx3cr1,Dock2,Fcer1g,Fcgr1,Fcgr2b,Fcgr3,H2-D1,H2-K1,H2-M3,H2-T23,Icam1,Ifih1,Ifitm3,Ighm,Il10rb,Inpp5d,Irf5,Jak3,Lcp1,Lgals9,Lyn,Myo1f,Ncf1,Oasl2,Plcg2,Ptpn6,Ptprc,Pycard,Rac2,Serping1,Syk,Tlr2,Tlr4,Tlr7,Tmem173,Tril,Tyrobp,Unc93b1,Vav1,Vsir,Zc3hav1

17 GO:0051707 response to another organism A 431/S 52 FDR 2.25E-22

Apobec1,B2m,Bcl3,Bst2,Cd180,Cd47,Cyba,Fcer1g,Fcgr1,Fcgr2b,Gbp2,Gbp3,Gbp6,Gbp7,Gbp9,H2-K1,H2-M3,H2-T23,Ifih1,Ifitm3,Ighm,Il10rb,Il17ra,Irf5,Irf8,Junb,Lgals9,Ltbr,Ly86,Lyn,Lyz2,Mrc1,Myo1f,Ncf1,Ncf2,Oasl2,Pik3cd,Plcg2,Ptgfr,Ptprc,Pycard,Stab1,Syk,Tlr2,Tlr4,Tlr7,Tmem173,Tnfrsf1a,Trem2,Unc93b1,Wipf1,Zc3hav1

26 GO:0001775 cell activation A 530/ S53 FDR 3.69E-19

Aif1,Apoe,B2m,Bcl3,Bcl6,Blnk,Cd180,Cd44,Cd47,Cd74,Cx3cr1,Dock2,Edn2,Fcer1g,Fcgr2b,Fcgr3,Fn1,H2-M3,H2-T23,Icam1,Ighm,Ikzf1,Il17ra,Inpp5d,Itgam,Itgb2,Jak3,Lcp1,Lgals9,Ltbr,Lyn,Myo1f,Nckap1l,Nfatc1,Nfkb2,P2ry12,Pik3cd,Plcg2,Ptpn6,Ptprc,Pycard,Rac2,Skap2,Syk,Tgfbr2,Timp1,Tlr2,Tlr4,Tlr7,Tyrobp,Vav1,Vcam1,Vsir

32 GO:0016064 immunoglobulin mediated immune response A 62/ S20 FDR 1.68E-16

Bcl3,Bcl6,C1qa,C1qb,C1qc,C1ra,C1s1,C3,C4b,Cd74,Cfi,Fcer1g,Fcgr1,Fcgr2b,Fcgr3,Ighm,Inpp5d,Ptpn6,Ptprc,Serping1

35 GO:0034097 response to cytokine A 349/ S40 FDR 3.58E-16

Bst2,Cd74,Cebpa,Csf1r,Csf2rb,Cx3cr1,Edn2,Fn1,Gbp2,Gbp3,Gbp6,Gbp7,Gbp9,H2-Q7,Ifitm3,Il13ra1,Il17ra,Il1r1,Il6ra,Irf5,Irgm2,Jak3,Junb,Mrc1,Nfkb2,Nlrc5,Osmr,Parp9,Ptpn6,Ptprc,Pycard,Serpina3n,Socs3,Stat3,Syk,Timp1,Timp2,Tmem173,Tnfrsf1a,Trem2

37 GO:0019882 antigen processing and presentation A 68/ S 20 FDR 1.20E-15

B2m,Cd74,Ctss,Fcer1g,Fcgr1,Fcgr2b,Fcgr3,H2-D1,H2-K1,H2-M3,H2-Q4,H2-Q7,H2-T23,Icam1,Ighm,Psmb8,Pycard,Rab32,Trem2,Unc93b1

39 GO:0048002 antigen processing and presentation of peptide antigen A38/ S 16 FDR 3.21E-15

B2m, Cd74,Ctss,Fcer1g,Fcgr1,Fcgr2b,Fcgr3,H2-D1,H2-K1,H2-M3,H2-Q4,H2-Q7,H2-T23,Pycard,Trem2,Unc93b1

40 GO:0002455 humoral immune response mediated by circulating immunoglobulin A 21/ S13 FDR 4.42E-15

Bcl3,C1qa,C1qb,C1qc,C1ra,C1s1,C3,Cfi,Fcgr2b,Ighm,Ptpn6,Ptprc,Serping1

49 GO:0042127 regulation of cell proliferation A 1024/ S 65 FDR 7.37E-14

Aif1,Apobec1,Apoe,Atf3,Bcl6,Btc,C3ar1,Cd109,Cd44,Cd47,Cd74,Cebpa,Csf1r,Cx3cr1,Cyba,Cyr61,Dab2,Edn2,Fcgr2b,Fgf2,Fn1,Gfap,Gpc3,Grn,H19,H2-M3,H2-T23,Hpgds,Hpse,Ifitm3,Ighm,Il6ra,Inpp5d,Jak3,Junb,Lgals9,Lif,Ltbr,Lyn,Marveld3,Muc2,Nckap1l,Nfatc1,Osmr,Ptgfr,Ptgs1,Ptpn6,Ptprc,Pycard,Rac2,Rtkn2,Skap2,Stat3,Syk,Tgfbr2,Tgm2,Timp1,Timp2,Tlr2,Tlr4,Tnfrsf1a,Trf,Vcam1,Vsir,Wwtr1

62 GO:0002757 immune response-activating signal transduction A147 S 24 FDR 7.14E-13

C3ar1,Cd180,Cmtm3,Cyba,Fcer1g,Ifih1,Ighm,Klhl6,Lgals9,Lyn,Nckap1l,Pik3ap1,Plcg2,Ptpn6,Ptprc,Rab7b,Skap2,Syk,Tlr2,Tlr4,Tlr7,Tril,Trim30a,Unc93b1

63 GO:0032103 positive regulation of response to external stimulus A147 S 24 FDR 7.14E-13

Aif1,C3,C3ar1,Cd180,Cd47,Cd74,Ctss,Cyba,Edn2,Fcer1g,Fcgr1,Fcgr3,Fgf2,Fn1,Il17ra,Lgals9,Ly86,Nckap1l,Pik3cg,Rac2,Tgm2,Tlr2,Tlr4,Tnfrsf1a

67 GO:0050727 regulation of inflammatory response A 168 S 25 FDR 1.65E-12

A2m,Apoe,Bcl6,C3,Cd44,Cd47,Ctss,Cx3cr1,Fcer1g,Fcgr1,Fcgr2b,Fcgr3,Il17ra,Il1r1,Lgals9,Lyn,Pik3ap1,Pik3cg,Pycard,Serping1,Socs3,Tgm2,Tlr2,Tlr4,Tnfrsf1a

71 GO:0006909 phagocytosis A 116 S 21 FDR 3.89E-12

Aif1,Bin2,C3,Cd47,Cyba,Dock2,Fcer1g,Fcgr1,Fcgr2b,Fcgr3,Ighm,Irf8,Itgb2,Nckap1l,Pld4,Pros1,Pycard,Syk,Tgm2,Trem2,Vav1

81 GO:0071310 cellular response to organic substance A 1250 S 68 FDR 1.73E-11

Atf3,B2m,Calca,Cd109,Cd180,Cd44,Cd68,Cd74,Cebpa,Csf1r,Csf2rb,Cx3cr1,Cyba,Cyr61,Dab2,Edn2,Fcer1g,Fgf2,Fn1,Gbp2,Gbp3,Gbp6,Gbp7,Gbp9,Gpc3,H2-Q7,Icam1,Ifitm3,Il13ra1,Il17ra,Il1r1,Il6ra,Irf5,Irf8,Irgm2,Itgb2,Jak3,Junb,Ly86,Lyn,Mrc1,Msn,Myof,Ncf2,Nlrc5,Osmr,P2ry12,P2ry13,Pik3cg,Ptgfr,Ptpn6,Ptprc,Pycard,Socs3,Spidr,Stat3,Syk,Tgfbr2,Timp2,Tlr2,Tlr4,Tmem173,Tnfrsf1a,Trem2,Ucp2,Vcam1,Vsir,Zc3hav1

93 GO:0007166 cell surface receptor signaling pathway A 1537 S 74 FDR 4.44E-10

Adgre1,Adgrg6,Atf3,Bag3,Bcl6,Btc,C3ar1,Cd109,Cd180,Cd22,Cd44,Cd53,Cd74,Cebpa,Cmtm3,Csf1r,Csf2rb,Cx3cr1,Cyr61,Dab2,Edn2,Fcer1g,Fcgr1,Fcgr2b,Fcgr3,Fgf2,Gpc3,Gpr34,H2-M3,Icam1,Ifitm3,Ighm,Il13ra1,Il1r1,Il6ra,Irf5,Itgam,Itgb2,Jak3,Klhl6,Kremen1,Lgals9,Lif,Ltbr,Ly86,Lyn,Myof,Nckap1l,Nlrc5,Osmr,P2ry12,Pik3cd,Plcg2,Plxnd1,Ptpn6,Ptprc,Pycard,S1pr2,Scube1,Siglec1,Socs3,Stat3,Syk,Tgfbr2,Timp1,Tlr2,Tlr4,Tnfrsf1a,Trem2,Trf,Tyrobp,Vav1,Vsir,Wwtr1

104 GO:0008284positive regulation of cell proliferation A 570 S 40 FDR 1.63E-09

Aif1,Atf3,Bcl6,Btc,C3ar1,Cd47,Cd74,Csf1r,Cx3cr1,Cyba,Cyr61,Edn2,Fgf2,Fn1,Gfap,Grn,H2-T23,Hpse,Ighm,Il6ra,Jak3,Lif,Lyn,Nckap1l,Osmr,Ptgfr,Ptpn6,Ptprc,Pycard,Rac2,Rtkn2,Stat3,Syk,Tgfbr2,Tgm2,Timp1,Tlr4,Trf,Vcam1,Wwtr1

107 GO:0042113 B cell activation A145 S 20 FDR 2.07E-09

Bcl3,Bcl6,Blnk,Cd180,Cd74,Fcgr2b,Ighm,Ikzf1,Inpp5d,Jak3,Lyn,Nckap1l,Nfatc1,Pik3cd,Plcg2,Ptpn6,Ptprc,Skap2,Syk,Tlr4

111 GO:0045785 positive regulation of cell adhesion A 239 S 25 FDR 3.12E-09

Aif1,Calca,Cd44,Cd47,Cd74,Cyr61,Dab2,Fn1,H2-M3,H2-T23,Icam1,Ikzf1,Jak3,Lgals9,Myo10,Nckap1l,Ptpn6,Ptprc,Pycard,Spp1,Syk,Tgfbr2,Tgm2,Vav1,Vcam1

114 GO:0002821 positive regulation of adaptive immune response A 53 S 13 FDR 3.49E-09

B2m,C3,Cd44,Cd74,Fcer1g,Fcgr1,Fcgr3,H2-D1,H2-K1,H2-M3,H2-T23,Ptprc,Pycard

116 GO:0002218 activation of innate immune response A 78 S 15 FDR 4.77E-09

Cd180,Cyba,Ifih1,Lgals9,Lyn,Pik3ap1,Pycard,Rab7b,Tlr2,Tlr4,Tlr7,Tmem173,Tril,Trim30a,Unc93b1

125 GO:1903039 positive regulation of leukocyte cell-cell adhesion A 96 S 16 FDR 9.44E-09

Aif1,Cd44,Cd47,Cd74,H2-M3,H2-T23,Icam1,Ikzf1,Jak3,Lgals9,Nckap1l,Ptprc,Pycard,Syk,Tgfbr2,Vcam1

127 GO:0030099myeloid cell differentiation A 254 S 25 FDR 9.87E-09

B2m,Bcl6,C1qc,Cd109,Cebpa,Clec2d,Csf1r,Dab2,Fli1,Gpc3,Ikzf1,Inpp5d,Irf8,Itgam,Junb,Lif,Ltbr,Lyn,Nckap1l,Nfatc1,Ptpn6,Rab7b,Tgfbr2,Trf,Tyrobp

134 GO:0034341response to interferon-gamma A 38 S 11 FDR 1.32E-08

Bst2,Gbp2,Gbp3,Gbp6,Gbp7,Gbp9,H2-Q7,Ifitm3,Mrc1,Nlrc5,Parp9

151 GO:0070372 regulation of ERK1 and ERK2 cascade A 160 S 19 FDR 6.35E-08

Atf3,C3,Cd44,Cd74,Csf1r,Cyr61,Dab2,Fgf2,Fn1,Glipr2,Icam1,Lif,Lyn,Ptpn6,Pycard,Syk,Tlr2,Tlr4,Trem2

156 GO:0032496response to lipopolysaccharide A 146 S 18 FDR 8.98E-08

B2m,Cd180,Gbp2,Gbp6,Irf8,Junb,Lgals9,Ltbr,Ly86,Lyn,Mrc1,Ncf2,Plcg2,Ptgfr,Pycard,Tlr4,Tnfrsf1a,Trem2

180 GO:0007155cell adhesion A 1059 S52 FDR 3.56E-07

Aif1,B2m,Bag3,Bcl3,Bcl6,Calca,Cd22,Cd33,Cd44,Cd47,Cd74,Cx3cr1,Cyr61,Dab2,Dock2,Fblim1,Fcer1g,Fn1,Gpnmb,H2-M3,H2-T23,Hpse,Icam1,Ikzf1,Itgam,Itgb2,Jak3,Lad1,Lcp1,Lgals3bp,Lgals9,Lyn,Micall2,Myo10,Myo1f,Nckap1l,P2ry12,Ptpn6,Ptprc,Pycard,Rac2,Siglec1,Spp1,Stab1,Syk,Tagln2,Tgfbr2,Tgm2,Vav1,Vcam1,Vsir,Zc3hav1

194 GO:0002718regulation of cytokine production involved in immune response A 32 S 9 FDR 5.32E-07

B2m,Bcl6,Cd74,Fcer1g,Jak3,Tlr2,Tlr4,Tril,Vsir

196 GO:0046633alpha-beta T cell proliferation A 16 S 7 FDR 6.70E-07

Cd44,Dock2,H2-T23,Lgals9,Ptprc,Syk,Vsir

204 GO:0032760positive regulation of tumor necrosis factor production A 45 S 10 FDR 9.76E-07

Cyba,Cybb,Fcer1g,Fcgr3,H2-T23,Lgals9,Pycard,Tlr2,Tlr4,Tnfrsf1a

213 GO:0002495 antigen processing and presentation of peptide antigen via MHC class II

A 11 S 6 FDR 1.28E-06 Cd74,Fcer1g,Fcgr2b,Pycard,Trem2,Unc93b1

225 GO:0008285 negative regulation of cell proliferation A 440 S29 FDR 2.10E-06

Aif1,Apoe,Bcl6,Cd109,Cd44,Cebpa,Csf1r,Dab2,Fcgr2b,Fgf2,Gpc3,H19,H2-M3,Hpgds,Ifitm3,Inpp5d,Lgals9,Lif,Lyn,Marveld3,Muc2,Nfatc1,Ptpn6,Skap2,Stat3,Tgfbr2,Timp2,Tlr2,Vsir

227 GO:0002429 immune response-activating cell surface receptor signaling pathway A 77 S 12

FDR 2.34E-06 C3ar1,Cmtm3,Fcer1g,Ighm,Klhl6,Lyn,Nckap1l,Plcg2,Ptpn6,Ptprc,Syk,Tlr2

228 GO:0030183B cell differentiation A 77 S 12 FDR 2.34E-06

Bcl3,Bcl6,Ighm,Ikzf1,Inpp5d,Jak3,Nckap1l,Nfatc1,Plcg2,Ptpn6,Ptprc,Syk

234 GO:0045597 positive regulation of cell differentiation A 695 S 38 FDR 2.46E-06

Apoe,Bcl6,Btc,Cd53,Cd74,Cebpa,Cebpd,Csf1r,Cx3cr1,Cyr61,Dab2,Fgf2,Fn1,Gfap,Glipr2,Grn,H2-M3,Ikzf1,Il1r1,Inpp5d,Junb,Lgals9,Lif,Lyn,Nckap1l,Ptprc,Rab7b,S1pr2,Socs3,Syk,Tgfbr2,Timp2,Tlr2,Trf,Vsir,Wwtr1,Xlr3b,Zbtb7c

266 GO:1902106 negative regulation of leukocyte differentiation A 59 S10 FDR 1.11E-05

Bcl6,C1qc,Cd44,Cd74,Clec2d,H2-M3,Inpp5d,Jak3,Lyn,Tmem176a

276 GO:0002275 myeloid cell activation involved in immune response A 48 S 9 FDR 1.70E-05

Cx3cr1,Dock2,Fcer1g,Lyn,Myo1f,Pycard,Rac2,Syk,Tyrobp

279 GO:0050731 positive regulation of peptidyl-tyrosine phosphorylation A 112 S 13 FDR 1.90E-05

Agrn,Cd44,Cd74,Csf1r,Icam1,Ighm,Il6ra,Lif,Lyn,Syk,Tlr4,Tnfrsf1a,Trem2

283 GO:0030335positive regulation of cell migration A 312 S 22 FDR 2.29E-05

Aif1,C3ar1,Cd74,Csf1r,Cyr61,Dab2,Edn2,Fgf2,Fn1,Glipr2,Icam1,Il1r1,Lgals9,Lyn,Myo1f,Nckap1l,Pik3cd,Ptprc,Pycard,Rac2,Tgfbr2,Tlr2

308 GO:0035456 response to interferon-beta A 29 S 7 FDR 4.53E-05

Bst2,Gbp2,Gbp3,Gbp6,Ifitm3,Irgm2,Tmem173

311 GO:0071222 cellular response to lipopolysaccharide A 87 S 11 FDR 5.21E-05

B2m,Cd180,Gbp2,Gbp6,Irf8,Ly86,Lyn,Mrc1,Pycard,Tlr4,Trem2

313 GO:0097028 dendritic cell differentiation A 20 S 6 FDR 6.12E-05

H2-M3,Ltbr,Lyn,Tgfbr2,Tmem176a,Trem2

314 GO:0050707 regulation of cytokine secretion A 72 S 10 FDR 6.34E-05

Csf1r,Fn1,Il17ra,Lgals9,Lyn,Pycard,Syk,Tlr2,Tlr4,Unc93b1

321 GO:0042108 positive regulation of cytokine biosynthetic process A 31 S 7 FDR 7.08E-05

Bcl3, Cybb,Fcgr3,Syk,Tlr2,Tlr4,Tlr7

335 GO:0045582 positive regulation of T cell differentiation A 45 S 8 FDR 9.02E-05

Cd74, H2-M3, Ikzf1,Lgals9,Nckap1l,Ptprc,Syk,Tgfbr2

341 GO:0042102 positive regulation of T cell proliferation A 46 S 8 FDR 0.000105932

Aif1, H2-T23, Jak3, Nckap1l,Ptprc,Pycard,Syk,Vcam1

345 GO:0015850organic hydroxy compound transport A 115 S 12 FDR 0.000128209

Abcc3, Apoe,Dab2,Fcer1g,Fcgr3,Ly6e,P2ry12,Ptgs1,Slc6a2,Slco2b1,Syk,Tlr2

349 GO:0045577 regulation of B cell differentiation A 23 S 6 FDR 0.000137301

Ikzf1, Inpp5d,Nckap1l,Ptpn6,Ptprc,Syk

363 GO:0045824 negative regulation of innate immune response A 25 S 6 FDR 0.00022278

A2m, Clec2d, H2-T23,Lgals9,Nlrc5,Serping1

377 GO:0045453bone resorption A 39 S 7 FDR 0.000312042

Csf1r, Ctss, Inpp5d,Rac2,Spp1,Syk,Trf

384 GO:0009620 response to fungus A 17 S 5 FDR 0.000402109

Il17ra, Ncf1, Pik3cd, Syk, Tlr2

385 GO:0042554superoxide anion generation A 17 S 5 FDR 0.000402109

Cyba,Cybb,Ncf1,Ncf2,Syk

387 GO:0007229 integrin-mediated signaling pathway A 56 S 8 FDR 0.000416925

Dab2, Fcer1g, Itgam, Itgb2,Syk,Timp1,Tyrobp,Vav1

391 GO:0002921negative regulation of humoral immune response A 9 S 4 FDR 0.000430345

A2m, Fcgr2b, Ptpn6, Serping1

393 GO:0050855regulation of B cell receptor signaling pathway A 9 S 4 FDR 0.000430345

Cmtm3, Lyn, Ptpn6,Ptprc

394 GO:0061081positive regulation of myeloid leukocyte cytokine production involved in immune response
A 9 S 4 FDR 0.000430345 Cd74,Fcer1g,Tlr2,Tlr4

397 GO:0002286T cell activation involved in immune response A 42 S 7 FDR 0.000482801

Bcl3,Bcl6,Fcer1g,H2-M3,Icam1,Jak3,Lcp1

400 GO:0032729 positive regulation of interferon-gamma production A 29 S 6 FDR 0.000514753

Bcl3, H2-M3,Irf8,Pycard,Tlr4,Tlr7

401 GO:0032845 negative regulation of homeostatic process A 134 S 12 FDR 0.000514753

Cd44, Cd74, Cyba, Fcer1g,Fcgr2b,Fgf2,Inpp5d,Lyn,Plcg2,Ptpn6,Ptprc,Ucp2

419 GO:0002283neutrophil activation involved in immune response A 10 S 4 FDR 0.00066226

Fcer1g, Myo1f,Syk,Tyrobp

420 GO:0034134toll-like receptor 2 signaling pathway A 10 S 4 FDR 0.00066226

Cyba, Lyn,Pik3ap1,Tlr2

421 GO:0034142toll-like receptor 4 signaling pathway A 19 S 5 FDR 0.00066226

Lyn, Pik3ap1,Rab7b,Tlr4,Tril

427 GO:0007186G-protein coupled receptor signaling pathway A 400 S 22 FDR 0.000794173

Adgre1,Adgrg6,Apoe,C3,C3ar1,Calca,Cx3cr1,Edn2,Gna14,Gnb3,Gpr34,Kctd12,P2ry12,P2ry13,Pik3cg,Ptgfr,Rac2, Rgs10,S1pr2,Syk,Tgm2,Vav1

434 GO:0032735 positive regulation of interleukin-12 production A 21 B 5 FDR 0.001099037

H2-M3, Irf8,Tlr2,Tlr4,Unc93b1

440 GO:0050715positive regulation of cytokine secretion A 49 S 7 FDR 0.001223082

Csf1r,Il17ra,Lgals9,Pycard,Syk,Tlr2,Unc93b1

446 GO:0001911 negative regulation of leukocyte mediated cytotoxicity A 12 S 4 FDR 0.001431616

Clec2d, H2-T23, Lgals9,Ptprc

447 GO:0002507tolerance induction A 12 S 4 FDR 0.001431616 C3ar1,H2-M3,Lyn,Tgfbr2

460 GO:0002833 positive regulation of response to biotic stimulus A 23 S 5 FDR 0.001599132

Cd180, Cyba,Lgals9,Ly86,Tlr2

461 GO:0035458 cellular response to interferon-beta A 23 S 5 FDR 0.001599132

Gbp2, Gbp3,Gbp6,Irgm2,Tmem173

462 GO:0007204positive regulation of cytosolic calcium ion concentration A 154 S12

FDR 0.001747672 C3ar1, Cyba,Edn2,Fgf2,Jak3,Lyn,Pik3cg,Plcg2,Ptgfr,Ptpn6,Ptprc,Tgm2

465 GO:0045576mast cell activation A 37 S 6 FDR 0.001894304 Fcer1g,Fcgr3,Lyn,Rac2,Syk,Tlr4

466 GO:0002467germinal center formation A 13 S 4 FDR 0.001894304 Bcl3,Bcl6,Klhl6,Nfkb2

477 GO:0032715negative regulation of interleukin-6 production A 25 S 5 FDR 0.002462463

Inpp5d,Nckap1l,Tlr4,Tnfrsf1a,Trim30a

484 GO:0002576platelet degranulation A 6 S 3 FDR 0.002708082 Fcer1g,Lyn,Syk

486 GO:0002890negative regulation of immunoglobulin mediated immune response A 6 S 3

FDR 0.002708082 Bcl6,Fcgr2b,Ptpn6

487 GO:0002923regulation of humoral immune response mediated by circulating immunoglobulin

A 6 S 3 FDR 0.002708082 Fcgr2b,Ptpn6,Ptprc

488 GO:0034162toll-like receptor 9 signaling pathway A 6 S 3 FDR 0.002708082

Pik3ap1,Rab7b,Unc93b1

490 GO:0032608interferon-beta production A 40 S 6 FDR 0.002708082

Ifih1, Pycard, Tlr2, Tlr4,Tlr7,Tmem173

499 GO:0001773myeloid dendritic cell activation A 15 S 4 FDR 0.003357545

Dock2, Ltbr, Pycard, Tgfbr2

500 GO:0045579 positive regulation of B cell differentiation A 15 S 4 FDR 0.003357545

Ikzf1, Inpp5d,Nckap1l,Syk

502 GO:0001782B cell homeostasis A 27 S 5 FDR 0.003357545 Cd44,Cd74,Lyn,Nckap1l,Pik3cd

503 GO:0010543regulation of platelet activation A 27 S 5 FDR 0.003357545

Apoe, Fcer1g,Lyn,Syk,Tlr4

505 GO:0045123cellular extravasation A 27 S 5 FDR 0.003357545 Icam1,Il1r1,Itgam,Itgb2,Vcam1

511 GO:0048535lymph node development A 16 S 4 FDR 0.004302724 Ikzf1,Jak3,Ltbr,Nfkb2

516 GO:0032490detection of molecule of bacterial origin A 7 S 3 FDR 0.004394846

Tlr2, Tlr4,Trem2

519 GO:0060907positive regulation of macrophage cytokine production A 7 S 3 FDR 0.004394846

Cd74, Tlr2,Tlr4

528 GO:0002312B cell activation involved in immune response A 45 S 6 FDR 0.004997966

Bcl3,Bcl6,Cd180,Plcg2,Ptprc,Tlr4

530 GO:0043066negative regulation of apoptotic process A 623 S 27 FDR 0.004997966

Aif1,Apoe,Bag3,Bcl3,Bcl6,Btc,Cd44,Cd74,Csf1r,Cx3cr1,Cyr61,Dab2,Fcer1g,Fcgr2b,Fn1,Icam1,Jak3,Mt1,Nckap1l,
Pik3cg,Ptgfr,Socs3,Spp1,Stat3,Timp1,Tnfrsf1a,Ucp2

532 GO:0045766positive regulation of angiogenesis A 83 S 8 FDR 0.005236187

C3,C3ar1,Cx3cr1,Cybb,Fgf2,Itgb2,Tgfbr2,Tnfrsf1a

535 GO:0045730respiratory burst A 17 S4 FDR 0.005236187 Cyba,Cybb,Ncf1,Rac2

542 GO:0070304 positive regulation of stress-activated protein kinase signaling cascade A 105 S 9

FDR 0.00544059 Dab2,Fgd2,Gadd45b,Ltbr,Lyn,Pycard,Syk,Tlr4,Trf

550 GO:0048661positive regulation of smooth muscle cell proliferation A 66 S 7 FDR 0.006427544

Aif1,C3ar1,Cyba,Fgf2,Il6ra,Tgfbr2,Tgm2

552 GO:0001820serotonin secretion A 8 S 3 FDR 0.006427544 Fcer1g,Fcgr3,Syk

553 GO:0006968cellular defense response A 8 S 3 FDR 0.006427544 B2m,Clec2d,Ncf1

554 GO:0032494response to peptidoglycan A 8 S 3 FDR 0.006427544 Irf5,Tlr2,Trem2

555 GO:0032736 positive regulation of interleukin-13 production A 8 S3 FDR 0.00642

H2-T23,Il17ra,Tlr4

557 GO:0045060 negative thymic T cell selection A 8 S 3 FDR 0.006427544 Cd74,Dock2,Ptprc

558 GO:0050832 defense response to fungus A 8 S 3 FDR 0.006427544 Il17ra,Ncf1,Pik3cd

561 GO:0071404 cellular response to low-density lipoprotein particle stimulus A 8 S 3 FDR 0.006427544

Fcer1g,Itgb2,Syk

562 GO:0097459 iron ion import into cell A 8 S 3 FDR 0.006427544 B2m,Steap4,Trf

567 GO:0050728 negative regulation of inflammatory response A 68 S 7 FDR 0.007671041
Apoe,Cd44,Cx3cr1,Fcgr2b,Lgals9,Socs3,Tnfrsf1a

568 GO:0046330 positive regulation of JNK cascade A 89 S 8 FDR 0.007787324

Dab2, Fgd2,Gadd45b,Ltbr,Pycard,Syk,Tlr4,Trf

569 GO:0002532 production of molecular mediator involved in inflammatory response A 33 S FDR 0.0078617

Fcer1g, Il17ra,Lyn,Syk,Tlr4

578 GO:0007565 female pregnancy A 70 S 7 FDR 0.008928028 A2m,Grn,H2-Q7,Junb,Lgals9,Lif,Timp1

579 GO:0031663 lipopolysaccharide-mediated signaling pathway A 34 S 5 0.72 FDR 0.009024345

Cd180,Ly86,Lyn,Tlr4,Trem2

580 GO:2001238 positive regulation of extrinsic apoptotic signaling pathway A 51 S 6 FDR 0.009024345

Atf3,Jak3,Ltbr,Ptprc,Pycard,Siglec1

582 GO:0032700 negative regulation of interleukin-17 production A 9 S 3 FDR 0.009152313

Nckap1l,Tlr2,Tlr4

585 GO:0046641 positive regulation of alpha-beta T cell proliferation A 9 S 3 FDR 0.009152313

H2-T23, Ptprc,Syk

590 GO:0001516 prostaglandin biosynthetic process A 20 S 4 FDR 0.009215

Cd74, Edn2,Hpgds,Ptgs1

595 GO:0042771 intrinsic apoptotic signaling pathway in response to DNA damage by p53 class mediator

A 35 S 5 FDR 0.009985485 Bcl3, Cd44,Cd74,Phlda3,Pycard

596 GO:0061045 negative regulation of wound healing A 35 S 5 FDR 0.009985485

Apoe, Cd109,Fgf2,Pros1,Serping1

597 GO:2000107 negative regulation of leukocyte apoptotic process A 35 S 5 FDR 0.009985485

Cd44,Cd74,Fcer1g,Fcgr2b,Jak3

604 GO:0043065 positive regulation of apoptotic process A 431 S 20 FDR 0.011210314

Agrn,Atf3,Bcl6,Cd44,Cyr61,Gadd45b,Inpp5d,Irf5,Jak3,Ltbr,Lyn,Muc2,Ncf2,Phlda3,Ptprc,Pycard,Siglec1,Tgm2,Tlr4,Tnfrsf1a

610 GO:0002281 macrophage activation involved in immune response A 10 S 3 FDR 0.012147722

Cx3cr1, Syk,Tyrobp

612 GO:0002719negative regulation of cytokine production involved in immune response A 10 S 3 FDR 0.012147722 Bcl6,Jak3,Vsir

613 GO:0006953acute-phase response A 10 S 3 FDR 0.012147722 Fn1,Serpina3n,Stat3

619 GO:2000778positive regulation of interleukin-6 secretion A 10 S 3 0.01214 Lgals9,Pycard,Unc93b1

622 GO:0032757positive regulation of interleukin-8 production A 22 S 4 FDR 0.012738816

Pycard, Tlr2,Tlr4,Tlr

625 GO:2000108positive regulation of leukocyte apoptotic process A 22 S 4 FDR 0.012738816

Cd44, Jak3,Lyn,Siglec1

633 GO:0001961positive regulation of cytokine-mediated signaling pathway A 23 S 4 FDR 0.01492

Cd74,Il1r1,Nlrc5,Trem2

637 GO:0043304regulation of mast cell degranulation A 23 S 4 FDR 0.014929168

Fcer1g, Lyn,Rac2,Syk

642 GO:0002755MyD88-dependent toll-like receptor signaling pathway A 11 S 3 FDR 0.015651323

Tlr2, Tlr4,Tlr7

646 GO:0042346positive regulation of NF-kappaB import into nucleus A 11 S 3 FDR 0.015651323

Tlr2, Tlr4,Tlr7

647 GO:0045059positive thymic T cell selection A 11 S 3 FDR 0.015651323

Cd74, Dock2, Ptprc

648 GO:0045589regulation of regulatory T cell differentiation A 11 S 3 FDR 0.015651323

Cd44, H2-M3, Lgals9

658 GO:0030225macrophage differentiation A 24 S 4 FDR 0.016926647 C1qc,Cebpa,Itgam,Lif

660 GO:0045581negative regulation of T cell differentiation A 24 S 4 FDR 0.016926647

Bcl6,Cd44,Cd74,Jak3

661 GO:0050798activated T cell proliferation A 24 S 4 FDR 0.0169 Itgam,Itgb2,Jak3,Pycard

664 GO:0000187activation of MAPK activity A 80 S 7 FDR 0.017097711Cd74,Dab2,Ighm,Ptprc,Syk,Tlr4,Trf

670 GO:0048260positive regulation of receptor-mediated endocytosis A 41 S 5 FDR 0.018485015

B2m,Dab2,Plcg2,Syk,Trf

676 GO:0045058T cell selection A 25 S 4 FDR 0.019277356 Cd74,Dock2,Ptprc,Syk

678 GO:0051353positive regulation of oxidoreductase activity A 25 S 4 FDR 0.019277356

Apoe,Cyba,Edn2,Lgals9

682 GO:0002717positive regulation of natural killer cell mediated immunity A 12 S 3 FDR 0.0192 H2-M3,H2-T23,Vav1

684 GO:0006910phagocytosis, recognition A 12 S 3 FDR 0.019277356 Fcgr1, Fcgr3,Ighm

685 GO:0007252I-kappaB phosphorylation A 12 S 3 FDR 0.019277356 Tlr2, Tlr4, Tlr7

686 GO:0007263nitric oxide mediated signal transduction A 12 S 3 FDR 0.019277 Apoe,Mt1,Mt2

687 GO:0015682ferric iron transport A 12 S 3 FDR 0.019277356 B2m,Steap4,Trf

690 GO:0030889negative regulation of B cell proliferation A 12 S 3 FDR 0.0192 Fcgr2b,Inpp5d,Lyn

691 GO:0032727positive regulation of interferon-alpha production A 12 S 3 FDR 0.0193 Ifih1,Tlr4,Tlr7

692 GO:0033630positive regulation of cell adhesion mediated by integrin A 12 S 3 FDR 0.0193 Nckap1l,Ptpn6,Syk

694 GO:0045780positive regulation of bone resorption A 12 S 3 FDR 0.01928 Spp1,Syk,Trf

699 GO:0010466negative regulation of peptidase activity A 106 S 8 FDR 0.0199044

A2m, Cd109,Cd44,Gpc3,Serpina3n,Serping1,Timp1,Timp2

712 GO:0015874norepinephrine transport A 13 S 3 FDR 0.023853612 P2ry12, Ptgs1, Slc6a2

713 GO:0019730antimicrobial humoral response A 13 S 3 FDR 0.02385 B2m, Bcl3, H2-T23

717 GO:2000406positive regulation of T cell migration A 13 S 3 FDR 0.02385 Aif1,Lgals9,Pycard

718 GO:0007566embryo implantation A 27 S 4 FDR 0.024504979 Grn,H2-Q7,Lif,Timp1

727 GO:0010575positive regulation of vascular endothelial growth factor production A 14 S 3 FDR 0.029

C3, C3ar1, Hpse

730 GO:0034122negative regulation of toll-like receptor signaling pathway A 14 S 3 FDR 0.029

Lyn, Rab7b,Trim30a

732 GO:0051209release of sequestered calcium ion into cytosol A 67 S 6 FDR 0.029769604

Cyba,Fgf2,Lyn,Plcg2,Ptpn6,Ptprc

734 GO:0002287alpha-beta T cell activation involved in immune response A 29 S 4 FDR 0.031393687 Bcl3, Bcl6,H2-M3,Jak3

739 GO:0045944positive regulation of transcription from RNA polymerase II promoter A 759 S 28 FDR 0.0318223Agrn,Atf3,Bcl3,Cebpa,Cebpd,Cyr61,Dab2,Fgf2,Fli1,Fosb,Hexb,Ikzf1,Irf5,Irf8,Jak3,Junb,Lif,Lpin3,Nfatc1,Nfkb2,Nlrc5,Stat3,Tlr2,Tlr4,Tmem173,Tnfrsf1a,Wwtr1,Zbtb7c

741 GO:0008360regulation of cell shape A 116 S 8 FDR 0.033029744 Csf1r, Fblim1,Fn1,Hexb,Icam1,Msn,Myo10,Wipf1

747 GO:0032720negative regulation of tumor necrosis factor production A 30 S 4 FDR 0.034 Bcl3,Gpnmb,Tlr4,Trim30a

753 GO:0051043regulation of membrane protein ectodomain proteolysis A 15 S 3 FDR 0.035 Apoe,Timp1,Timp2

760 GO:0010718positive regulation of epithelial to mesenchymal transition A 31 S 4 FDR 0.038744063 Dab2, Glipr2,Tgfbr2,Wwtr1

767 GO:0001778 plasma membrane repair A 5 S 2 FDR 0.040466379 Dysf, Myof

769 GO:0002645positive regulation of tolerance induction A 5 S 2 FDR 0.0405 H2-M3, Tgfbr2

770 GO:0002664regulation of T cell tolerance induction A 5 S 2 FDR 0.040466379 H2-M3,Tgfbr2

772 GO:0032667regulation of interleukin-23 production A 5 S 2 FDR 0.0404664 Il17ra,Tlr4

773 GO:0032762mast cell cytokine production A 5 S 2 FDR 0.040466379 Bcl6,Fcer1g

774 GO:0034135regulation of toll-like receptor 2 signaling pathway A 5 S 2 FDR 0.0405 Cyba,Lyn

775 GO:0042270protection from natural killer cell mediated cytotoxicity A 5 S 2 FDR 0.04047 Clec2d, H2-T23

776 GO:0045359positive regulation of interferon-beta biosynthetic process A 5 S 2 FDR 0.0405 Tlr4,Tlr7

777 GO:0046689response to mercury ion A 5 S 2 FDR 0.0405 Fn1, Gatm

778 GO:0050861positive regulation of B cell receptor signaling pathway A 5 S 2 FDR 0.0405 Cmtm3,Lyn

779 GO:0060100positive regulation of phagocytosis, engulfment A 5 S 2 0.0405 Fcgr1, Nckap1l

780 GO:0061687detoxification of inorganic compound A 5 S 2 FDR 0.0405 Mt1, Mt2

781 GO:0070627ferrous iron import A 5 S 2 FDR 0.0405 B2m, Trf

785 GO:2000668regulation of dendritic cell apoptotic process A 5 S 2 FDR 0.0405 Jak3,Lyn

786 GO:2001179regulation of interleukin-10 secretion A 5 S 2 FDR 0.0405 Lgals9, Pycard

787 GO:2001198regulation of dendritic cell differentiation A 5 S 2 FDR 0.0405 H2-M3, Tmem176a

789 GO:0042517positive regulation of tyrosine phosphorylation of Stat3 protein A 16 S 3 FDR 0.0405 Csf1r,Il6ra,Lif

793 GO:0070373negative regulation of ERK1 and ERK2 cascade A 51 S 5 FDR 0.041275763 Atf3,Dab2,Lif,Lyn,Tlr4

800 GO:0045646regulation of erythrocyte differentiation A 33 S 4 FDR 0.04653575

B2m, Inpp5d,Lyn,Nckap1l

805 GO:0048246macrophage chemotaxis A 17 S 3 FDR 0.047528795 C3ar1,Cx3cr1,Edn2
