# Supplementary figures and images for: Genetic Rescue of X-Linked Retinoschisis Mouse (Rs1−/y) Retina Induces Quiescence of the Retinal Microglial Inflammatory State Following AAV8-RS1 Gene Transfer and Identifies Gene Networks Underlying Retinal Recovery
Source: Hum Gene Ther. 2021 Jul 16;32(13-14):667–81. doi: 10.1089/hum.2020.213 (PMC8312029; doi:10.1089/hum.2020.213)

**Figure S2. Differentially Expressed Genes**

**P21-Rs1-KO vs. WT**

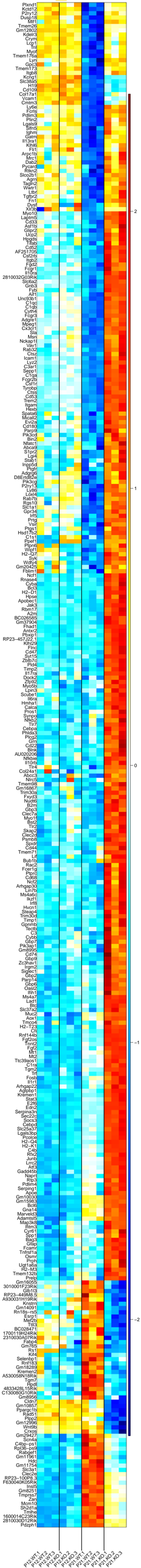

Supplement: Supplemental data [file Supp_Fig2.pdf]
