# Supplementary material for: Genetic Rescue of X-Linked Retinoschisis Mouse (Rs1−/y) Retina Induces Quiescence of the Retinal Microglial Inflammatory State Following AAV8-RS1 Gene Transfer and Identifies Gene Networks Underlying Retinal Recovery
Source: Hum Gene Ther. 2021 Jul 16;32(13-14):667–81. doi: 10.1089/hum.2020.213 (PMC8312029; doi:10.1089/hum.2020.213)
Supplement: Supplemental data [file Supp_Fig3.pdf]

Figure S3. Gene expression changes in P21 ***Rs1***-KO retina (qRT-PCR validation)

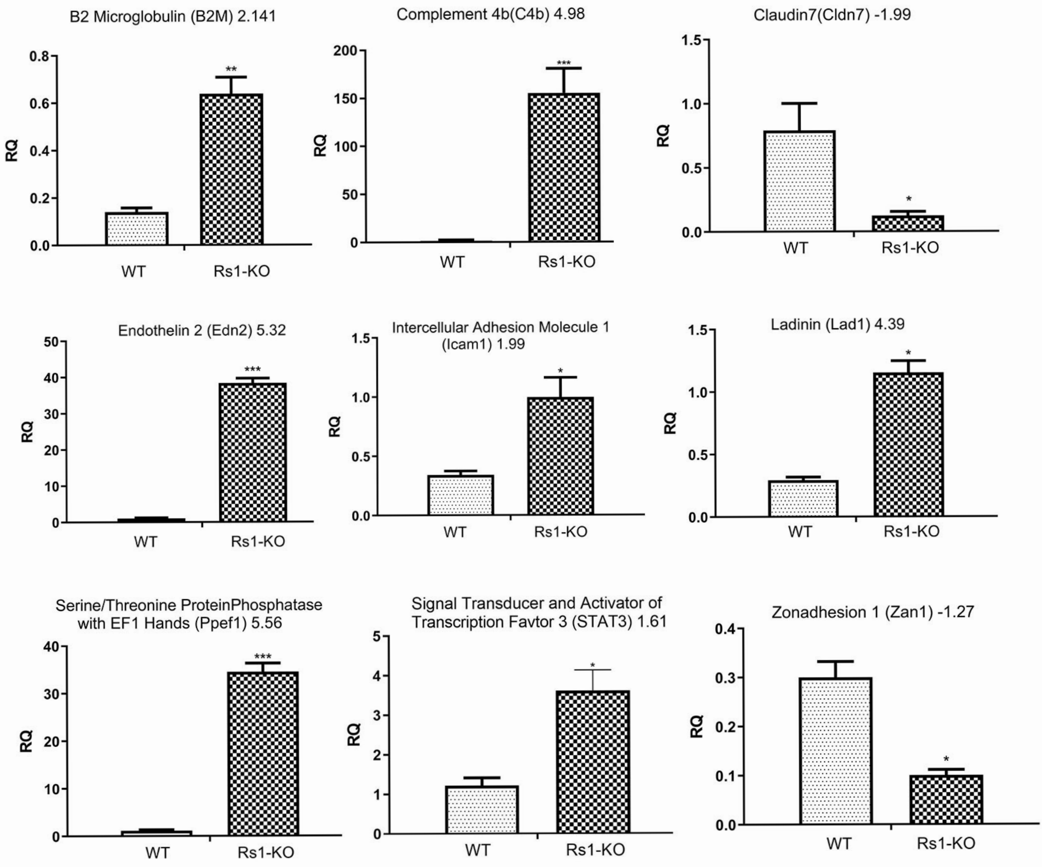

qRT-PCR confirmation of common RNA-Seq changes between WT and *Rs1*-KO mice retinas. TaqMan mRNA assay reactions were run in triplicate in three independent experiments on the QuantStudio 6 and 7 Flex Real-Time PCR System with TaqMan® probes (Thermo Fisher Scientific). 18s rRNA was amplified as endogenous control (internal reference). The results were expressed as n-fold induction or inhibition in gene expression relative to endogenous control calculated using the ΔΔCT method. The data was processed using Quant Studio™ 6 and 7 Flex Real-Time PCR System Software. The data expressed as Mean ± SEM. Differences between groups were analyzed two-stage step-up method of Benjamini, Krieger and Yekutieli: * $p<0.05$; ** $p<0.001$; **** $p<0.0001$.
