# Supplementary material for: Genetic Rescue of X-Linked Retinoschisis Mouse (Rs1−/y) Retina Induces Quiescence of the Retinal Microglial Inflammatory State Following AAV8-RS1 Gene Transfer and Identifies Gene Networks Underlying Retinal Recovery
Source: Hum Gene Ther. 2021 Jul 16;32(13-14):667–81. doi: 10.1089/hum.2020.213 (PMC8312029; doi:10.1089/hum.2020.213)
Supplement: Supplemental data [file Supp_Table5.pdf]

Table S5. Selected list of differentially expressed genes *Rs1*-KO vs. WT at P21

Gene symbol	Description	Mouse Genome Informatics ID[a]	Log2 FC[b]	P.Value	adj.P.Value[c]
	**MICROGLIA**				
Aif1	allograft inflammatory factor 1	MGI:1343098	2.34	2.07E-05	1.01E-03
Apoe	apolipoprotein E	MGI:88057	1.02	1.27E-04	2.77E-03
Tgfbr2	transforming growth factor, beta receptor II	MGI:98729	1.33	5.34E-05	1.71E-03
Cybb	cytochrome b-245, beta polypeptide	MGI:88574	2.82	4.20E-05	1.49E-03
Clec7a	C-type lectin domain family 7, member a	MGI:1861431	4.32	3.52E-06	3.14E-04
Csf1r	colony stimulating factor 1 receptor	MGI:1339758	2.34	3.49E-08	2.63E-05
Cx3cr1	chemokine (C-X3-C motif) receptor 1	MGI:1333815	2.21	5.30E-08	2.67E-05
Fcrls	Fc receptor-like S, scavenger receptor	MGI:1933397	3.68	2.28E-08	2.53E-05
Gpr34	G protein-coupled receptor 34	MGI:1346334	2.18	9.78E-07	1.55E-04
Gpnmb	glycoprotein (transmembrane) nmb	MGI:1934765	2.94	1.69E-05	8.98E-04
Hexb	hexosaminidase B	MGI:96074	1.42	2.87E-07	8.08E-05
Il10ra	interleukin 10 receptor, alpha	MGI:96538	2.30	9.92E-07	1.56E-04
P2ry13	purinergic receptor P2Y, G-protein coupled 13	MGI:1921441	1.78	1.92E-05	9.72E-04
P2ry12	purinergic receptor P2Y, G-protein coupled 12	MGI:1918089	1.03	2.47E-04	3.98E-03
Siglec1	sialic acid binding Ig-like lectin 1, sialoadhesin	MGI:99668	1.42	2.19E-04	3.72E-03
Spp1	secreted phosphoprotein 1	MGI:98389	2.19	3.78E-04	5.00E-03
Trem2	triggering receptor expressed on myeloid cells 2	MGI:1913150	3.30	1.81E-08	2.08E-05
	**The inflammatory response to cell death**				
Bcl3	B cell leukemia/lymphoma 3	MGI:88140	5.05	3.53E-07	8.77E-05
C1qa	complement component 1, q subcomponent alpha	MGI:88223	2.61	8.00E-09	1.17E-05
C1qb	complement component 1, q subcomponent, beta	MGI:88224	2.75	2.11E-08	2.20E-05
C1qc	complement component 1, q subcomponent, C	MGI:88225	2.91	6.05E-08	2.85E-05
C3	complement component 3	MGI:88227	2.40	6.45E-05	1.9E-03
C4b	complement component 4B	MGI:88228	4.98	7.95E-10	2.90E-06
C3ar1	complement component 3a receptor 1	MGI:1097680	4.68	1.89E-06	2.22E-04
Edn2	endothelin 2 [	MGI:95284	5.32	8.40E-11	5.19E-07
Il6ra	interleukin 6 receptor, alpha	MGI:105304	2.01	8.21E-06	5.48E-04
Il10ra	interleukin 10 receptor, alpha	MGI:96538	2.29	9.92E-07	1.55E-04
Il17ra	interleukin 17 receptor A	MGI:107399	1.35	2.08E-06	2.37E-04
Nlrc5	NLR family, CARD domain containing 5	MGI:3612191	2.83	5.82E-05	1.80E-03
	**Innate Immunity**				
Tlr2	toll-like receptor 2	MGI:1346060	2.15	7.22E-05	2.00E-03
Tlr4	toll-like receptor 4	MGI:96824	1.36	4.9E-04	5.90E-03
Tlr9	toll-like receptor 7	MGI:2176882	3.56	1.92E-04	3.48-E03
	**Cluster of differentiation (CD) antigens**				
Cd33	CD33 antigen	MGI:99440	2.80	1.84E-05	9.51E-04
Cd52	Cd52	MGI:1346088	2.50	1.76E-05	9.20E-04
Cd53	CD53 antigen	MGI:88341	2.86	5.01E-08	2.63E-05
Cd68	CD68 antigen	MGI:88342	3.17	1.32E-05	7.59E-04
Cd74	antigen (MHC class II antigen-associated	MGI:96534	2.99	4.2E-04	5.3E-03
Cd180	CD180 antigen	MGI:1194924	2.82	5.16E-06	4.10E-04
	**Extracellular Matrix Degradation and Remodeling in Development and Disease**				
Adamtsl5	adamts-like 5	MGI:1913798	1.05	3.59E-04	4.9E-03
Fgf2	fibroblast growth factor 2	MGI:95516	3.14	1.17E-07	4.27E-05
Timp1	tissue inhibitor of metalloproteinase 1	MGI:98752	2.20	6.45E-05	1.9E-03
Timp2	tissue inhibitor of metalloproteinase 2	MGI:98753	1.00	2.41E-06	2.5E-04
	**Other cell surface molecules**				
Fcer1g	Fc receptor, IgE, high affinity I, gamma polypeptide	MGI:95496	2.17	1.27E-05	7.46E-04
Itgb2	integrin beta 2	MGI:96611	2.71	5.07E-06	4.06E-04
Icam1	intercellular adhesion molecule 1	MGI:96392	1.99	1.09E-05	6.72E-04
Lgals9	lectin, galactose binding, soluble 9	MGI:109496	1.43	9.83E-05	2.38E-04
Rab7b	Rab7b, member RAS oncogene family	MGI:2442295	2.04	1.28E-05	7.47E-04
Sla	src-like adaptor	MGI:104295	1.17	7.32E-07	1.35E-04
Vcam1	vascular cell adhesion molecule 1	MGI:98926	2.76	9.44E-06	6.10E-04

Zan	zonadhesin	MGI:106656	-1.28	3.29E-06	3.03E-04
	**TMEM132: A new family of neural adhesion molecule**				
Tmem26	transmembrane protein 26	MGI:2143537	1.25	7.73E-05	2.01E-03
Tmem71	transmembrane protein 71	MGI:2146049	1.93	6.88E-05	2.00E-03
Tmem98	transmembrane protein 98	MGI:1923457	1.31	7.29E-05	2.00E-03
Tmem132b	transmembrane protein 132B	MGI:3609245	1.07	1.25E-04	2.76E-03
Tmem173	transmembrane protein 173 [	MGI:1919762	1.32	2.10E-04	3.66E-03
Tmem176a	transmembrane protein 176A	MGI:1913308	1.10	1.55E-05	8.51E-04
	**Cytokine Signaling-Transcription Factors**				
Atf3	activating transcription factor 3	MGI:109384	1.79	2.68E-06	2.66E-04
Cx3cr1	chemokine (C-X3-C motif) receptor 1	MGI:1333815	2.21	5.30E-08	2.67E-05
Fosb	FBJ osteosarcoma oncogene B	MGI:95575	1.70	1.77E-4	3.33E-03
Irf5	interferon regulatory factor 5	MGI:1350924	1.58	4.76E-05	1.59E-03
Irf8	interferon regulatory factor 8	MGI:96395	1.78	5.63E-05	1.77E-03
Jak3	Janus kinase 3	MGI:99928	2.85	4.76E-08	2.63E-05
Junb	jun B proto-oncogene	MGI:96647	1.33	8.88E-07	1.49E-04
Il6ra	interleukin 6 receptor, alpha	MGI:105304	2.01	8.21E-6	5.48E-04
P2ry12	purinergic receptor P2Y, G-protein coupled 12	MGI:1918089	1.03	2.46E-04	3.97E-03
Stat3	signal transducer and activator of transcription 3	MGI:103038	1.62	6.35E-07	1.11E-04

Abbreviations: a-MGI-Mouse Genome Informatics; b-log2FC-fold change expressed as log2 base; c-adj.P.Value: p value adjusted using Benjamini Hochberg method implemented in edgeR
