# Supplementary material for: Genetic Rescue of X-Linked Retinoschisis Mouse (Rs1−/y) Retina Induces Quiescence of the Retinal Microglial Inflammatory State Following AAV8-RS1 Gene Transfer and Identifies Gene Networks Underlying Retinal Recovery
Source: Hum Gene Ther. 2021 Jul 16;32(13-14):667–81. doi: 10.1089/hum.2020.213 (PMC8312029; doi:10.1089/hum.2020.213)
Supplement: Supplemental data [file Supp_Fig4.pdf]

Figure S4. Transgene *RS1* induced gene expression changes G7 vs. C7 and G35 vs. C35 retinas (q RT-PCR confirmation)

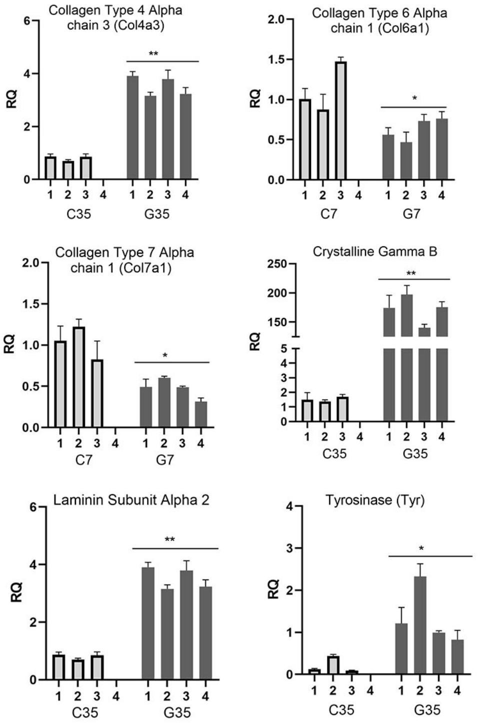

The experimental conditions are described in Figure S3.
* p<0.05; ** p<0.001
