# Supplementary material for: Genetic Rescue of X-Linked Retinoschisis Mouse (Rs1−/y) Retina Induces Quiescence of the Retinal Microglial Inflammatory State Following AAV8-RS1 Gene Transfer and Identifies Gene Networks Underlying Retinal Recovery
Source: Hum Gene Ther. 2021 Jul 16;32(13-14):667–81. doi: 10.1089/hum.2020.213 (PMC8312029; doi:10.1089/hum.2020.213)
Supplement: Supplemental data [file Supp_Table6.pdf]

Table S 6. Complete List of Significantly Differentially Expressed Gene (DEGs) List. G7 vs. C7 (*Rs1*-KO-AAV8-RS1 vs. *Rs1*-KO-AAV8-Null at post injection day 7)

Gene	Transcript Count	log FC	log CPM	P. Value	FDR
Mfsd10	21	-0.6466	3.806755	5.18E-07	3.82E-05
Col6a1	2	-1.00548	5.733852	1.77E-23	7.49E-20
Cfp	2	-0.64075	3.702949	1.14E-07	1.01E-05
Tcirg1	13	-0.82201	3.402773	6.57E-10	1.41E-07
Spag5	8	-0.60989	6.198976	5.12E-10	1.14E-07
Pon1	4	1.047961	3.908572	0.0012	0.019421
Akap8l	1	-0.5955	6.370557	1.70E-08	1.97E-06
Irf3	13	-0.69845	5.596849	8.02E-14	5.65E-11
Car11	5	-0.74434	4.684609	1.21E-10	3.12E-08
Nqo1	1	0.692777	2.779307	9.34E-06	0.000427
Mettl17	16	-0.63213	4.914896	3.93E-13	2.49E-10
Stxbp2	15	-0.59427	5.35059	3.83E-09	6.52E-07
Tyr	3	1.017343	4.257609	7.61E-07	5.14E-05
Dgkq	11	-0.75819	4.521357	5.62E-11	1.62E-08
Bcan	6	-0.73075	2.624759	3.62E-07	2.80E-05
Rasa4	13	-0.71025	5.099791	4.44E-14	3.76E-11
Tyrp1	4	0.873355	5.533343	1.22E-05	0.000516
Fpgs	10	-0.68455	2.974727	3.39E-08	3.47E-06
Taz	22	-0.69504	6.154963	1.23E-13	8.18E-11
Rnf112	7	-0.63089	4.279835	3.46E-09	6.19E-07
Dennd6b	6	-0.79319	6.578113	3.04E-17	3.85E-14
Cltrn	3	0.991324	3.704652	1.62E-05	0.000647
Col20a1	5	-0.61992	4.690585	2.04E-11	7.83E-09
Pipox	3	-0.62768	2.413175	2.68E-05	0.000989
Etv4	12	-0.7172	2.299034	6.64E-07	4.71E-05
Syne4	17	-1.25453	2.645041	1.74E-20	4.42E-17
Smpdl3a	3	0.609292	3.244342	0.000234	0.005396
Smtn	10	-0.58776	2.909681	5.32E-05	0.001697
Coro6	6	-0.59527	2.549055	2.33E-05	0.000882
Adam11	9	-0.59834	5.682396	3.86E-09	6.52E-07
Dct	4	0.838739	7.021643	0.000406	0.008372
Carmil3	12	-0.59749	5.697834	1.95E-07	1.63E-05
Trmu	9	-0.59376	4.348969	4.11E-09	6.86E-07
Enpp2	9	0.672736	7.012827	0.000511	0.009965
Cldn1	2	0.831158	3.96611	0.000211	0.004978
Aifm3	6	-1.02147	2.805262	3.16E-11	1.03E-08
Clic6	2	0.754984	4.357311	0.00017	0.004224
Gps2	5	-0.68731	4.793138	9.03E-13	4.98E-10

Rgs11	11	-1.06195	6.238859	2.00E-25	1.27E-21
Adamts10	12	-0.62562	4.736761	3.38E-06	0.000176
Col11a2	8	-0.92678	3.973882	4.14E-06	0.000208
Vps51	10	-0.73983	3.542695	2.66E-11	9.43E-09
Rps6kb2	11	-0.5969	3.758842	1.16E-05	0.000502
Doc2g	4	-0.71112	2.524208	2.26E-06	0.000129
Map4k2	22	-0.64356	4.968698	5.76E-09	8.21E-07
Col17a1	4	-0.66164	3.522819	6.73E-05	0.002029
Lrrc45	5	-0.73405	5.138772	2.75E-11	9.43E-09
Hps1	9	-0.67166	2.943284	4.60E-06	0.000229
Alas2	4	1.077035	3.181115	0.000188	0.004606
Itgb8	3	0.648534	6.719774	4.94E-05	0.001603
Pmel	3	1.10072	5.182524	1.17E-05	0.000502
Col7a1	8	-1.40898	4.866466	3.46E-19	6.27E-16
Rhbdl1	3	-0.62933	3.315575	1.51E-08	1.85E-06
Spopl	6	0.627493	4.344876	1.19E-05	0.000509
Ermn	1	0.998777	3.345858	0.00116	0.018937
Col5a1	2	-0.65677	4.058636	4.10E-07	3.11E-05
Notch1	11	-0.59794	5.957196	1.21E-05	0.000512
Arrdc1	12	-0.59454	2.743461	0.000203	0.004829
Cdc25b	7	-0.79201	3.095343	3.95E-06	0.0002
Ampd2	10	-0.69201	6.050799	5.73E-13	3.46E-10
Col11a1	7	-0.70196	4.3696	5.86E-06	0.000285
Lrat	1	1.005592	4.667314	0.001536	0.023368
Adam15	13	-0.67808	4.979713	9.07E-11	2.45E-08
Thbs3	11	-0.87547	2.50863	4.97E-06	0.000244
Rpe65	3	1.048371	5.658503	0.001855	0.027053
Ccdc163	14	-0.63003	4.81215	1.11E-12	5.87E-10
Sema3c	5	0.849873	3.694835	0.000849	0.014878
Miip	8	-0.63034	4.650082	1.18E-10	3.12E-08
P2rx2	6	-0.7808	2.234974	4.64E-07	3.44E-05
Gm20605	1	-0.89561	6.253866	4.63E-12	2.18E-09
Pon3	6	0.62468	2.408495	0.000454	0.009122
Gpnmb	5	0.623396	5.955434	0.000603	0.011424
Oca2	6	0.95785	2.627381	0.000111	0.003032
Cln3	19	-0.64703	4.714408	1.45E-08	1.80E-06
Cpxm2	5	0.869641	4.231233	0.000172	0.004266
Cacna1f	11	-0.67791	8.443478	1.22E-06	7.73E-05
Zmym3	18	-0.76086	6.926187	4.66E-10	1.06E-07
Flna	15	-0.66816	7.147688	2.27E-08	2.44E-06
Plxna3	9	-0.8042	4.727981	5.62E-11	1.62E-08
Dock6	8	-0.75477	3.570279	1.78E-05	0.000699
Man2c1	23	-0.72761	5.718804	2.95E-16	3.40E-13
Car12	6	1.124159	3.915901	0.000294	0.006484

Col6a4	4	-1.05117	2.756552	3.95E-15	3.86E-12
Slc16a8	2	0.91719	3.666944	0.000555	0.010696
Cpsf1	20	-0.60387	6.208659	1.86E-11	7.36E-09
Ssh3	2	-0.60117	4.173718	1.73E-07	1.47E-05
Mfrp	6	0.590917	5.47568	0.003916	0.046781
Tle6	14	-0.59398	3.601157	2.42E-06	0.000135
Tle2	16	-0.96523	5.051988	6.71E-14	5.00E-11
Cpne7	5	-0.67203	4.10899	6.57E-09	9.26E-07
Leng8	10	-0.70625	8.490044	2.38E-06	0.000134
Dok3	3	-0.59673	2.977119	7.03E-05	0.002109
Snapc4	14	-0.7587	5.027828	6.18E-13	3.56E-10
Cenpt	10	-0.6518	3.789997	1.61E-08	1.88E-06
Slc6a20a	3	1.077082	3.959879	2.77E-05	0.001019
Slc39a12	3	0.709278	2.890012	2.88E-05	0.001049
Tns2	9	-0.60244	4.021233	9.71E-07	6.39E-05
BC030499	6	-0.6555	6.994181	3.58E-09	6.21E-07
Slc16a9	2	0.894145	3.476427	1.04E-08	1.38E-06
Asic3	5	-0.95746	5.136069	7.81E-26	9.90E-22
Rtel1	21	-0.89654	5.295476	8.73E-23	2.77E-19
Ciz1	22	-0.66028	6.026837	1.07E-08	1.40E-06
Tbce	13	-0.5902	5.93944	5.30E-11	1.62E-08
Tspan10	2	0.773804	3.271151	0.000141	0.003664
Abcc8	8	-0.70049	4.059813	2.38E-07	1.93E-05
Thbs1	2	0.886904	5.494896	7.73E-05	0.002271
Sec1	1	-0.74177	2.769915	1.19E-06	7.60E-05
Slc26a7	4	0.766452	4.051476	0.000145	0.003741
Bace2	4	0.642144	3.42272	0.000705	0.012869
Slc16a11	11	-0.7111	2.407928	5.50E-07	4.01E-05
Rgl2	12	-0.81043	5.350904	5.95E-14	4.72E-11
Kank3	6	-0.71434	3.738779	1.86E-08	2.11E-06
Ccdc84	12	-0.59325	4.196262	3.54E-09	6.21E-07
Col27a1	7	-0.74741	4.156447	1.02E-11	4.47E-09
Mtmr11	7	-0.99975	3.694086	6.26E-18	9.93E-15
Serinc4	6	-0.59552	5.62082	2.97E-12	1.45E-09
Atg16l2	17	-0.59564	4.081562	4.35E-08	4.19E-06
Tpcn2	8	-0.71898	2.648486	4.22E-07	3.19E-05
Gemin4	2	-0.70387	2.855254	0.000144	0.003732
2310061I04Rik	9	-0.59008	4.827695	5.19E-09	7.90E-07
Hbb-bs	3	1.18465	8.277281	0.000301	0.006569
Lgals4	7	-0.82042	3.262563	7.36E-11	2.03E-08
Tdrd9	6	-0.99294	4.439259	2.56E-17	3.61E-14
Pld5	6	0.6095	2.793612	9.43E-05	0.002658
Izumo4	13	-0.71318	3.61917	1.02E-09	2.11E-07
Tmem150a	5	-0.66378	2.400188	1.74E-05	0.000686

Col8a2	2	0.929066	4.099155	0.001823	0.026731
Snx32	1	-0.78134	5.664611	1.03E-19	2.17E-16
Ccdc189	5	-0.63909	2.870253	2.99E-07	2.36E-05
Kcnt1	15	-0.89631	4.986778	3.13E-14	2.83E-11
Csf2ra	1	-0.70679	4.664171	7.10E-12	3.21E-09
Gckr	7	-0.71251	7.553238	6.70E-09	9.33E-07
Arr3	7	-0.78574	7.724154	3.91E-10	9.19E-08
Clasrp	13	-0.60121	5.358459	4.43E-07	3.32E-05
Cdh3	1	0.612679	2.707834	0.000667	0.012424
Ttr	1	1.108086	8.004403	0.000757	0.013635
Ppox	8	-0.63959	5.778142	1.79E-11	7.32E-09
Hdac10	8	-0.66253	4.464293	1.60E-08	1.87E-06
Col8a1	2	1.118309	5.479979	3.52E-06	0.00018
Hba-a2	2	1.100906	7.758885	0.000842	0.014849
Hba-a1	2	1.115803	7.062873	0.000699	0.012828
Slc39a2	2	-0.59622	2.90792	0.000218	0.005106
Acrbp	7	-0.68757	4.000099	1.98E-08	2.22E-06
4933439C10Rik	6	-0.64766	3.788055	1.48E-07	1.29E-05
Zfp414	7	-0.84852	4.556946	1.14E-15	1.20E-12
Crygb	1	2.789127	6.516208	0.003685	0.044985
Hbb-bt	1	1.182445	5.855703	0.000194	0.004705
Trim68	5	-0.69068	3.148427	3.57E-07	2.78E-05
Nrbp2	10	-0.77731	5.599613	2.72E-11	9.43E-09
Dcdc2b	3	-0.80553	2.219396	5.28E-08	4.96E-06
Kcnj13	2	0.903534	3.177849	0.00101	0.017065
Med12	9	-0.58966	6.359936	1.24E-08	1.59E-06
Pisd-ps1	4	-0.90887	3.58509	3.75E-05	0.001286
Slc2a4rg-ps	2	-0.75541	3.643976	4.31E-10	9.95E-08
Gm15612	1	-0.61731	2.743698	2.26E-06	0.000129
Snhg20	7	-0.65033	4.689371	1.22E-09	2.49E-07
Tgfbr3l	5	-0.60764	4.757124	2.42E-06	0.000135
AI480526	7	-0.67029	4.876582	2.64E-09	4.85E-07
Neat1	4	-0.67512	5.878173	4.89E-09	7.73E-07
Gm20507	1	-0.70568	2.916329	4.36E-08	4.19E-06
Mir5125	1	-0.60487	2.67326	0.00068	0.012604
AC149090.1	2	-0.89221	7.737431	2.17E-07	1.77E-05
Gm21750	1	0.58904	2.499844	0.000161	0.004077
Gm3764	11	-0.60497	3.748561	3.53E-06	0.00018
Gm26852	1	-0.66275	2.943725	1.55E-06	9.40E-05
Mirg	12	-0.819	3.098135	3.53E-08	3.55E-06
Rps6kb2	11	-0.5969	3.758842	1.16E-05	0.000502
Gm10925	1	0.858512	9.612805	1.56E-08	1.86E-06
Gm28437	1	1.68546	8.010629	2.40E-11	8.97E-09
Tmem150a	5	-0.66378	2.400188	1.74E-05	0.000686

Gm36995	1	-0.84389	3.944769	6.83E-10	1.44E-07
Gm38102	2	-0.63874	4.578794	1.40E-06	8.62E-05
Gm37422	1	-0.61709	2.704996	3.30E-06	0.000173
AY036118	1	1.306503	6.064245	5.38E-11	1.62E-08
4932422M17Rik	1	-0.81136	4.489757	6.04E-10	1.32E-07
CT010467.1	2	0.900463	9.207282	2.10E-07	1.73E-05
Map4k2	22	-0.64356	4.968698	5.76E-09	8.21E-07
Gm44597	2	-0.95455	3.627414	1.64E-10	4.17E-08
Gm49320	1	-0.78762	3.373718	1.02E-05	0.000455
Gm35339	1	-0.71245	2.827859	7.98E-08	7.28E-06
Gm49396	4	-0.68409	4.700452	2.00E-09	3.74E-07
Flt3l	13	-0.92132	2.724658	3.95E-11	1.25E-08
Gm48796	1	-0.59281	4.134897	1.30E-08	1.64E-06
