# Supplementary material for: Genetic Rescue of X-Linked Retinoschisis Mouse (Rs1−/y) Retina Induces Quiescence of the Retinal Microglial Inflammatory State Following AAV8-RS1 Gene Transfer and Identifies Gene Networks Underlying Retinal Recovery
Source: Hum Gene Ther. 2021 Jul 16;32(13-14):667–81. doi: 10.1089/hum.2020.213 (PMC8312029; doi:10.1089/hum.2020.213)
Supplement: Supplemental data [file Supp_Table7.pdf]

Table S 7. Complete List of Significantly Differentially Expressed Gene (DEGs) List. G35 vs. C35 (*Rs1*-KO-AAV8-*RS1* vs. *Rs1*-KO-AAV8-Null at post injection day 35)

Gene	Transcript Count	log FC	log CPM	P. Value	FDR
Ccnd2	8	0.875595	4.350793	0.000636	0.040276
S100a4	2	1.424992	3.152079	6.58E-05	0.009169
Col6a1	2	-0.63213	5.704914	1.2E-08	1.38E-05
Car11	5	-0.73643	5.150183	1.76E-10	3.19E-07
Cavin1	2	0.615154	3.911563	0.000327	0.025864
Tyr	3	1.456981	3.053254	6.93E-07	0.000333
Tyrp1	4	0.99175	4.630351	0.000578	0.037572
Carhsp1	1	0.815937	4.820578	6.81E-05	0.00928
Gprc5b	4	0.587076	5.992193	1.92E-06	0.000677
Slc16a12	1	0.885838	2.366052	0.000418	0.030817
Ptgds	5	0.894975	8.268942	0.000103	0.012154
Cltrn	3	1.080625	2.853902	6.95E-06	0.001834
Pltp	7	0.797907	4.430455	5.41E-05	0.007958
Pmp22	5	0.883077	4.792388	1.62E-07	0.000103
Sparc	9	0.630827	8.189131	0.000109	0.012725
Dalrd3	9	-0.68202	6.485688	1.04E-06	0.00044
Krt10	2	-0.72697	2.892253	0.000128	0.014117
Perp	1	1.387551	2.718889	2.17E-08	2.3E-05
Lama2	8	1.216257	3.022615	7.84E-05	0.010237
Pygl	6	0.669451	4.974679	6.75E-05	0.00928
Ogn	1	1.435382	3.698257	3.54E-09	5.61E-06
Rgr	6	1.286973	6.28221	0.000659	0.041215
Emp2	2	0.924855	3.148185	2.09E-05	0.003893
Fstl1	6	0.660788	5.822822	0.000106	0.012436
Slc15a2	14	0.78564	5.831404	0.000217	0.019967
Clic6	2	0.827507	4.181877	0.000116	0.01329
P3h3	7	-0.61495	5.669111	5.38E-07	0.000284
Crim1	1	1.146564	6.741473	4.4E-08	4.28E-05
Vit	4	1.276832	5.171959	7.09E-07	0.000333
Rgs11	11	-0.64189	6.309126	1.13E-08	1.38E-05
Lama3	2	0.920276	2.951253	0.00029	0.024188
Pdgfrb	2	0.666265	4.065963	0.00022	0.020011
Anxa1	7	1.221052	5.642087	1.99E-06	0.000677
Rdh5	7	0.890461	4.740003	0.000592	0.038305
Pmel	3	0.866942	4.815687	0.000212	0.019644
Larp1b	15	0.840115	3.021974	0.000343	0.02649
Igfbp5	2	0.735774	6.260951	0.00029	0.024188
Kif26b	3	0.755174	3.872405	3.38E-05	0.005419
Vim	6	0.670693	7.928247	0.000498	0.034087
Nek6	5	0.615461	3.712828	0.000512	0.034316

Gsn	7	1.240574	4.16846	2.03E-06	0.000677
Lhx3	4	-0.63962	3.692659	0.000506	0.034166
Neb	13	-0.70642	2.522229	0.000371	0.02853
Dapl1	2	1.248719	5.682816	9.66E-07	0.000422
Itga6	8	0.892655	4.362032	5.08E-05	0.007759
Bmp2	1	0.999934	2.397067	0.000118	0.01329
Bfsp1	2	3.585823	4.949586	0.000424	0.030871
Fabp5	2	1.856232	5.955337	1.11E-07	8.81E-05
Mme	8	1.576769	3.439984	0.000685	0.041438
Hmgcs2	2	1.478666	3.355062	9.42E-07	0.000422
Wls	7	1.390072	4.233961	8.2E-11	2.08E-07
Hspg2	3	1.862662	5.664995	0.000535	0.035513
Tinagl1	9	1.362991	2.068345	7.07E-08	5.97E-05
Htra3	6	1.051466	3.124075	0.000504	0.034166
Fosl2	3	0.68176	3.284625	0.000299	0.024446
Pdgfra	9	0.77721	4.828766	6.81E-08	5.97E-05
Fkbp9	2	0.635686	4.551537	9.52E-05	0.011414
Gpnmb	5	1.079079	5.104554	0.000695	0.041438
Nupr1	6	0.963909	5.572226	0.000692	0.041438
Fgfr2	27	1.313603	3.409697	1.88E-05	0.003646
Col4a6	3	1.15976	3.542001	0.000542	0.035588
Col4a5	4	0.895176	5.556488	0.000145	0.015363
Rs1	3	0.636885	6.006876	1.54E-05	0.00336
Slc7a3	7	-0.79167	2.399803	0.000221	0.020037
Rab11fip1	6	0.717402	3.415543	0.000331	0.025864
Sfrp1	1	2.031401	5.321899	9.28E-05	0.011414
Slc7a2	5	0.818782	5.725658	4.34E-07	0.000239
Stra6	14	0.975416	4.421221	0.00087	0.049493
Frrs1	6	1.116677	2.288308	1.21E-07	8.99E-05
Ucp2	11	0.832828	4.497592	0.000135	0.014718
Rfc3	7	-0.61773	3.231112	0.000463	0.032243
Loxl2	2	0.7877	3.76915	0.000507	0.034166
Flvcr2	2	1.366487	2.472938	5.43E-05	0.007958
Mfrp	6	1.088587	4.902194	4.17E-06	0.001288
Tle6	14	-0.73089	3.984842	3.02E-05	0.005023
Tle2	16	-0.59711	5.34633	1.55E-06	0.000579
Ror1	1	0.978777	2.418085	1.62E-05	0.003372
Acta2	1	1.771043	3.239059	2.02E-06	0.000677
Igfbp7	2	1.791254	6.977775	0.000833	0.048454
Arsi	1	1.795087	3.079993	5.75E-05	0.008285
Etl4	20	0.832018	4.69281	8.13E-06	0.002021
Bend3	4	0.870262	2.86373	0.000516	0.034443
Asic3	5	-0.66047	5.520154	9.36E-09	1.32E-05
Abhd2	2	0.62382	5.949067	7.83E-06	0.002014

Ppl	2	1.240951	3.439982	0.000621	0.039733
Nhsl1	9	1.049884	3.152184	5.46E-05	0.007958
Slc26a7	4	0.961177	3.372841	1.31E-05	0.002966
Slc16a11	11	-0.62284	3.465594	0.000692	0.041438
Fhdc1	5	1.672539	4.033024	3.27E-13	2.07E-09
Kcnj12	3	0.616015	3.754733	0.000286	0.024188
Ccdc84	12	-0.62799	4.377209	1.73E-05	0.00353
Aqp5	6	1.552179	2.285866	3.04E-06	0.000987
Dock5	3	1.465167	4.365629	0.000416	0.030815
Tent5c	3	1.707033	3.667609	3.3E-06	0.001045
Erich5	1	1.370776	4.191488	3.49E-05	0.005524
Cd24a	3	2.108078	6.224945	7.8E-05	0.010237
Lix1	2	1.429071	3.678766	0.000788	0.046441
Sox1ot	2	1.023465	2.912549	0.00086	0.049357
Tmem72	2	0.699867	4.711575	0.000459	0.032243
Gja1	8	0.873282	5.687688	0.000491	0.033835
Ezr	2	0.958629	5.150256	3.21E-05	0.005217
Aldh1a1	6	0.830474	6.380069	1.58E-07	0.000103
Phgdh	4	1.168636	4.501814	4.29E-07	0.000239
Shisa6	3	1.151956	3.983161	5.83E-12	1.85E-08
Maf	2	0.818348	5.001559	1.9E-05	0.003646
Slc35f3	1	0.747944	2.255037	0.000612	0.039377
Gstm1	4	0.794676	5.516594	0.000297	0.02444
Kcnh1	3	0.677263	4.142419	2.96E-05	0.005023
Tcea2	8	-0.78573	3.93262	1.04E-05	0.002544
Cdh3	1	0.864262	2.256428	0.000461	0.032243
Ica1	12	-0.60251	4.117759	0.000398	0.030209
Hist2h2aa1	1	0.892648	3.410808	0.000444	0.031783
mt-Co2	1	0.643013	10.01836	0.000632	0.040268
Col4a4	2	1.247731	5.223459	4.69E-06	0.001382
Ahnak	2	0.744111	5.794069	1.14E-10	2.4E-07
Fat1	6	1.175013	5.984619	0.000647	0.040802
Serpinh1	7	0.619602	4.341833	0.000697	0.041438
Zscan18	6	-0.58603	4.288557	0.000158	0.016435
Crygb	1	5.018712	6.350948	0.000837	0.048454
Ccdc61	8	0.7337	2.705953	0.000682	0.041438
Nynrin	3	0.75302	4.478868	4.63E-06	0.001382
Ass1	4	0.603848	5.492838	1.24E-05	0.002857
Cpt1b	4	-1.2609	3.605194	2.98E-07	0.00018
R3hdml	1	1.775673	3.30115	0.000162	0.016435
Sap25	6	-1.09731	2.569563	2.33E-05	0.004162
Col4a3	4	1.546554	5.359349	1.6E-05	0.003372
C1qtnf5	8	0.779707	3.105164	0.000232	0.020594
Gm12895	1	0.819966	7.757375	0.000163	0.016435

Gm12896	1	0.819966	7.757375	0.000163	0.016435
Snhg20	7	-0.69686	5.60356	1.33E-06	0.000527
Mir5125	1	-1.03786	2.826832	0.000319	0.025754
Gm3764	11	-0.62102	4.15238	6.58E-05	0.009169
Mir6236	1	0.722186	11.10446	0.000835	0.048454
Gm10925	1	-1.99534	8.445811	9.55E-18	1.21E-13
Gm28437	1	-1.14024	9.740975	1.29E-07	9.07E-05
Gm37904	1	-0.76215	3.326684	0.000174	0.017211
Gm36995	1	-0.59032	3.744142	0.000449	0.031974
Gm12895	1	0.819966	7.757375	0.000163	0.016435
Gm49396	4	-0.61916	4.594396	7.95E-06	0.002014
Flt3l	13	-0.67146	3.160217	0.000676	0.041438
Gm38431	2	1.589261	2.178895	2.92E-12	1.23E-08
Gm49327	3	-0.70469	4.071349	6.58E-06	0.001774
Col4a6	3	1.15976	3.542001	0.000542	0.035588
