# Supplementary material for: Genetic Rescue of X-Linked Retinoschisis Mouse (Rs1−/y) Retina Induces Quiescence of the Retinal Microglial Inflammatory State Following AAV8-RS1 Gene Transfer and Identifies Gene Networks Underlying Retinal Recovery
Source: Hum Gene Ther. 2021 Jul 16;32(13-14):667–81. doi: 10.1089/hum.2020.213 (PMC8312029; doi:10.1089/hum.2020.213)
Supplement: Supplemental data [file Supp_Table8.pdf]

Table S8. Selected list of significantly differentially expressed gene in *Rs1*-KO retina after AAV8-*RS1* gene transfer.

Gene symbol	Description	Mouse Genome Informatics [a]	Log 2-Fold Change [b]	P Value	Adj P Value [c]
	**Collagens**				
Col5a1	collagen, type V, alpha 1	MGI:88457	-0.66	4.10E-07	3.11E-05
Col6a1	collagen, type VI, alpha 1 MGI:88459	MGI:88459	-1.01	1.77E-23	7.49E-20
Col6a4	collagen, type VI, alpha 4	MGI:1915803	-1.05	3.95E-15	3.86E-12
Col7a1	collagen, type VII, alpha 1	MGI:88462	-1.41	3.46E-19	6.27E-16
Col11a1	collagen, type XI, alpha 1	MGI:88446	-0.71	5.86E-06	2.84E-04
Col11a2	collagen, type XI, alpha 2	MGI:88447	-0.93	4.14E-06	2.08E-04
Col17a1	collagen, type XVII, alpha 1	MGI:88450	-0.66	6.73E-05	2.02E-03
Col20a1	collagen, type XX, alpha 1	MGI:1920618	-0.62	2.04E-11	7.83E-09
Col27a1	collagen, type XXVII, alpha 1	MGI:2672118	-0.75	1.02E-11	4.47E-09
	**Vascular Collagens**				
Col8a1	collagen, type VIII, alpha 1	MGI:88463	1.12	3.52E-06	1.80E-04
Col8a2	collagen, type VIII, alpha 2	MGI:88464	0.93	1.8E-03	2.67E-02
	**Basement Membrane Components**				
Col4a3	collagen, type IV, alpha 3	MGI:104688	1.55	1.60E-05	3.37E-04
Col4a4	collagen, type IV, alpha 4	MGI:104687	1.25	4.69E-06	1.38E-03
Col4a5	collagen, type IV, alpha 5	MGI:88456	0.89	1.45E-04	1.54E-03
Col4a6	collagen, type IV, alpha 6	MGI:2152695	1.16	5.41E-04	3.56E-03
Col6a1	collagen, type VI, alpha 1	MGI:88459	-0.63	1.20E-08	1.38E-05
Hspg2	perlecan (heparan sulfate proteoglycan 2)	MGI:96257	1.86	5.35E-04	3.55-E03
Lama2	laminin, alpha 2	MGI:99912	1.22	7.84E-05	1.02E-03
Lama3	laminin alpha3	MGI:99909	0.92	2.90E-05	2.42e-03
	**ECM**				
Bmp2	bone morphogenetic protein 2	MGI:88177	1.00	1.18E-04	1.32E-03
Fgfr2	fibroblast growth factor receptor 2	MGI:95523	1.31	1.88E-05	3.64E-02
Itga6	integrin alpha 6	MGI:96605	0.89	5.08E-05	7.75E-03
Lox2	lysyl oxidase-like 2	MGI:2137913	0.79	5.07E-04	3.41E-03
Ror1	receptor tyrosine kinase-like orphan receptor1	MGI:1347520	0.98	1.62E-05	3.33E-03
Serpinh1	serine (or cysteine) peptidase inhibitor, clade H, member 1	MGI:88283	0.62	6.97E-04	4.14E-02
	**Retinoid Cycle**				
Lrat	lecithin-retinol acyltransferase(	MGI:1891259	1.01	1.53E-03	2.33E-03
Rbp1	retinol binding protein 1, cellular	MGI:97876	0.61	4.44E-03	0.13
Rdh5	retinol dehydrogenase 5	MGI:1201412	0.97	8.69E-04	4.94E-03
Rpe65	retinal pigment epithelium 65	MGI:98001	1.05	1.85E-03	2.71E-03
Stra6	stimulated by retinoic acid gene 6	MGI:107742	0.97	8.69E-04	4.94E-03
	**Melanogenesis**				
Dct	dopachrome tautomerase	MGI:102563	0.84	1.22E-05	5.15E-04
Oc2	oculocutaneous albinism II	MGI:97454	0.96	1.11E-04	3.03E-03
Tyr	tyrosinase	MGI:98880	1.02	7.61E-07	5.14E-05
Tyrp1	tyrosinase-related protein 1	MGI:98881	0.87	1.22E-05	5.15E-04
	**Apoptosis**				
Aifm3	apoptosis-inducing factor, mitochondrion-3	MGI:1919418	-1.02	3.16 E-11	1.03E-08
	**Camera type eye development**				
Aqp5	aquaporin 5	MGI:106215	1.55	3.04E-06	9.86E-04
Aldh1a1	aldehyde dehydrogenase family 1, A1	MGI:1353450	0.83	1.58E-07	1.03E-04

Bfsp1	beaded filament structural protein 1,in lens	MGI:101770	3.58	4.23E-04	3.08E-02
Crygb	crystallin, gamma B	MGI:88522	5.01	8.37E-04	4.84E-02
Fat1	FAT atypical cadherin 1	MGI:109168	1.18	6.47E-04	4.08E-03
Gja1	gap junction protein, alpha 1	MGI:95713	0.87	4.91E-04	3.38E-02
Maf1	avian musculoaponeurotic fibrosarcoma oncogene homolog	MGI:96909	0.82	1.90E-05	3.64E-03
Mfrp	membrane frizzled-related protein	MGI:2385957	1.09	4.17E-06	1.28E-02
Pdgfra	platelet derived growth factor receptor, alpha polypeptide	MGI:97530	0.78	6.81E-08	5.97E-05
Pdgfrb	platelet derived growth factor receptor, beta polypeptide	MGI:97531	0.67	2.19E-04	2.00E-02
Vim	vimentin	MGI:98932	0.67	4.97E-04	3.40E-02
	**Adhesion**				
Anxa1	annexin A1	MGI:96819	1.22	1.99E-06	6.76E-02
Ass1	argininosuccinate synthetase 1	MGI:88090	0.60	1.24E-05	2.85E-03
Cdh3	cadherin 3	MGI:88356	0.86	4.60E-04	3.22E-03
Cd24a	CD24a antigen	MGI:88323	2.10	7.80E-05	1.02E-02
Dock5	dedicator of cytokinesis 5	MGI:2652871	1.47	4.15E-04	3.08E-02
Emp2	epithelial membrane protein 2	MGI:1098726	0.89	5.08E-05	7.76E-03
Ezr	ezrin	MGI:98931	0.96	3.21E-05	5.21E-03
Gpnmb	glycoprotein (transmembrane) nmb	MGI:1934765	1.08	6.95E-04	4.14E-02
Gsn	gelsolin	MGI:95851	1.24	2.03E-06	6.76E-04
Itga6	integrin alpha 6	MGI:96605	0.89	5.08E-05	7.76E-03
Igfbp5	insulin-like growth factor binding protein 5	MGI:96440	0.74	2.92E-04	2.41E-03
Igfbp7	insulin-like growth factor binding protein 7	MGI:1352480	1.79	8.32E-04	4.82E-03
Kif26b	kinesin family member 26B	MGI:2447076	0.75	3.38E-05	5.41E-03
Pdgfra	platelet derived growth factor receptor, α	MGI:97530	0.78	6.81E-08	5.97E-05
Shisa6	shisa family member 6	MGI:2685725	1.15	5.83E-12	1.85E-08
Sfrp1	secreted frizzled-related protein 1	MGI:892014	2.03	9.28E-05	1.11E-02
Tinagl1	tubulointerstitial nephritis antigen-like 1	MGI:2137617	1.36	7.07E-08	5.97E-05
Vit	vitrin	MGI:1921449	1.28	7.09E-07	3.33E-03

a-MGI-Mouse gene ID; **b**-log2FC-fold change expressed as log2 base; **c**-adj.P.Value: p value adjusted using Benjamini Hochberg method implemented in edgeR
